# Mechanosensory Piezo2 regulated by gut microbiota participates in the development of visceral hypersensitivity and intestinal dysmotility

**DOI:** 10.1080/19490976.2025.2497399

**Published:** 2025-04-28

**Authors:** Haonan Zheng, Yuzhu Chen, Siqi Lu, Zuojing Liu, Yinchao Ma, Cunzheng Zhang, Yiming Zhang, Jindong Zhang, Chang Liu, Ming Chu, Fei Pei, Shuangjiang Liu, Liping Duan

**Affiliations:** a Department of Gastroenterology, Peking University Third Hospital, Beijing, P. R. China; b Beijing Key Laboratory for *Helicobacter pylori* Infection and Upper Gastrointestinal Diseases, Peking University Third Hospital, Beijing, P. R. China; c PKUMed-EKEMed Joint Laboratory for Human Microbiome Research, Peking University Health Science Center, Beijing, P. R. China; d Department of Immunology, NHC Key Laboratory of Medical Immunology, School of Basic Medical Sciences, Peking University, Beijing, P. R. China; e State Key Laboratory of Microbial Technology, Shandong University, Qingdao, P. R. China; f Department of Pathology, Peking University Third Hospital; School of Basic Medical Sciences, Peking University, Beijing, P. R. China; g State Key Laboratory of Microbial Resources, Institute of Microbiology, Chinese Academy of Sciences, Beijing, P. R. China

**Keywords:** Piezo2, *Fusobacterium varium*, visceral hypersensitivity, gut dysmotility

## Abstract

The gut microbiota plays a crucial role in the manifestation of intestinal dysfunction associated with irritable bowel syndrome (IBS). The mechanosensory Piezo2 has been implicated in the regulation of intestinal function. However, it remains unclear whether Piezo2 is modulated by the gut microbiota, thus contributing to the development of visceral hypersensitivity and gut dysmotility. The study enrolled patients with diarrhea-predominant IBS (IBS-D) alongside healthy controls (HC). Questionnaires, rectal barostat test, and colonoscopy with mucosal biopsy were conducted. Fecal microbiota transplantation (FMT) was performed using samples from HC or IBS-D patients, and interventions with *Akkermansia muciniphila* or *Fusobacterium varium* were carried out on colon- or dorsal root ganglion (DRG)- Piezo2 knockdown pseudo-germ-free mice. Visceral sensitivity and intestinal motility were assessed. Piezo2 levels were detected using western blot and immunofluorescence. Fecal 16S rRNA sequencing and cecum untargeted metabolomics analysis, followed by molecular docking predictions of Piezo2, were also performed. The ratio of Piezo2^+^/5-HT^+^ cells was lower in IBS-D patients, positively correlated with visceral sensation and intestinal dysbiosis. The mice that received FMT from IBS-D patients exhibited colonic dysmotility and visceral hypersensitivity, along with elevated Piezo2 protein levels in the colon and DRG. Knockdown of Piezo2 in the colon or DRG ameliorated the FMT-induced colonic dysmotility and visceral hypersensitivity. Fecal 16S rRNA sequencing revealed distinct microbiota composition. Notably, *Fusobacterium varium*, but not *Akkermansia muciniphila*, induced gut dysmotility and visceral hypersensitivity, effects that could be alleviated by colon or DRG Piezo2 knockdown. Additionally, *Fusobacterium varium* lead to increased Piezo2 protein levels, as well as elevated levels of indole-3-acetic acid and indole-3-acrylic acid, which were predicted to bind to Piezo2, causing disturbances. Piezo2 can be regulated by gut microbiota and involved in visceral hypersensitivity and colonic dysmotility, with *Fusobacterium varium* playing a crucial role.

## Graphical abstract

**Figure:**
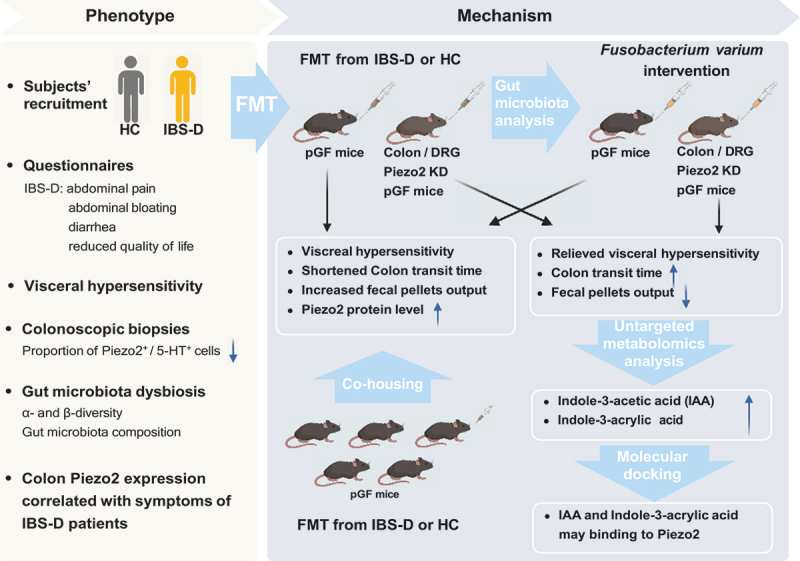

## Introduction

Irritable bowel syndrome (IBS) is the most typical disorders of gut-brain interaction (DGBIs), characterized by chronic abdominal pain accompanied by alterations in bowel habits.^1^ The intestinal microbiota exerts a profound influence on the mechanisms underlying IBS.^2,3^ It is well-established that the intestinal microbiota interacts with the nervous system, thereby modulating visceral sensitivity and gastrointestinal (GI) motility.^4–6^ One notable effect of this interaction is the regulation of serotonin, also known as 5-hydroxytryptamine (5-HT), which is released from enterochromaffin cells (ECs).

Piezo2 is a mechanosensory ion channel that plays a crucial role in sensing mechanical stimuli in various cells and tissues throughout the body, including the GI tract.^7–9^ Although studies suggest a potential connection between Piezo2 and visceral hypersensitivity,^10–13^ as well as GI motility,^14,15^ its precise role remains elusive. Given that Piezo2 mediates the release of 5-HT from ECs in response to mechanical stimulation,^8,9^ its significance becomes increasingly apparent. Furthermore, related research has demonstrated that Piezo1, a molecule belonging to the same family as Piezo2, can be modulated by intestinal microbiota-produced single-stranded RNA (ssRNA), thereby affecting 5-HT production.^4^ Therefore, it is worthwhile to investigate whether Piezo2 is influenced by the gut microbiota and to determine its specific contribution to visceral hypersensitivity and GI dysmotility. Identifying the key bacterial strains involved and elucidating the underlying mechanisms deserve comprehensive exploration.

## Materials and methods

### Human

#### Subjects’ recruitment

Patients with diarrhea predominant-IBS (IBS-D) who met the ROME III criteria were recruited from the Department of Gastroenterology at Peking University Third Hospital. Additionally, healthy control (HC) volunteers without any GI symptoms were recruited through advertisement. All participants were aged between 18 and 65 years. The exclusive criteria were as follows: (1) having taken antibiotics, probiotics/prebiotics, or psychotropic medications in the preceding four weeks; (2) suffering from systemic or GI diseases such as diabetes mellitus and inflammatory bowel disease; (3) currently affected by infectious diseases of the respiratory, digestive, or urinary system; or (4) having a history of abdominal surgery. Detailed information about the participants is provided in Supplementary Table S1.

For enrolled patients with IBS-D, to exclude any organic colonic diseases and microscopic colitis, all participants either underwent colonoscopy or had undergone colonoscopy/barium enema in the six months prior. A totally of 55 IBS-D patients and 19 HC were enrolled in this study.

Demographic data, including age, gender, and body mass index (BMI), were recorded. The participants’ quality of life and overall health were assessed using the 36-item Short-Form (SF-36).^16^ Daily bowel movement frequency and stool consistency were evaluated using the Bristol Stool Form Scale (BSFS).^17^ The severity of GI symptoms was measured using both the Gastrointestinal Symptom Rating Scale (GSRS)^18^ and the IBS Symptom Severity Scores (IBS-SSS).^19^ Visceral sensitivity was assessed through a rectal distension procedure utilizing the BAROSTAT (Distender Series II; G&J Electronics, Ontario, Canada), which allowed for the determination of individual sensory thresholds related to initial sensation, desire to defecate, urge need to defecate and pain perception.

Mucosal biopsies were obtained from the sigmoid colon during colonoscopy and immediately fixed in a 10% formalin solution, followed by embedding in paraffin for preservation. Stool samples were self-collected by the participants, promptly placed in stool nucleic acid collection and preservation tubes (#45660, Norgen Biotek), transported at room temperature, and then stored at  − 80°C for subsequent gut microbiota analysis.

## Ethical approval

All participants voluntarily enrolled in this study and furnished their written informed consent. The study adhered to the principles outlined in the *Declaration of Helsinki* and obtained approval from the Medical Science Research Ethics Committee of Peking University Third Hospital (approval number: 2013–12).

### Animals

#### Mice

Eight-week-old male C57BL/6J mice were procured from SPF (Beijing) Biotechnology Co., Ltd., and maintained under specific pathogens free (SPF) conditions. Housed in SPF facilities with a 12-hour light-dark cycle, the mice underwent a one-week acclimation period prior to the experiment. Unless otherwise specified, all mice had *ad libitum* access to sterilized food and water.

A total of 268 mice were utilized in this study. Among them, 156 mice were employed for fecal microbiota transplantation (FMT) experiments, specifically: 60 for FMT alone, 24 for co-housing experiments, 32 for FMT following colon Piezo2 knockdown, and 40 for FMT after dorsal root ganglion (DRG) Piezo2 knockdown. The remaining 112 mice were used for single bacteria intervention experiments, specifically: 40 for *Akkermansia muciniphila*/*Fusobacterium varium* intervention, 32 for *Fusobacterium varium* intervention after colon Piezo2 knockdown, and 40 for *Fusobacterium varium* intervention following DRG Piezo2 knockdown.

Following the experiment, all mice were anesthetized with 2,2,2-tribromoethanol at a dosage of 0.2 ml per 10 g of body weight via intraperitoneal injection. Tissue samples were collected after cervical dislocation and immediately stored at  − 80°C.

All experimental procedures were approved by the Animal Welfare and Ethics Committee of Peking University (approval number: PUIRB-LA2023152).

### Antibiotics (Abx) treatment to establish pseudo-germ-free (pGF) mice

To establish pGF mice, we administered an antibiotic cocktail as described in a previous study.^20^ Briefly, SPF mice received twice-daily oral gavage of a mixture containing neomycin (100 mg/kg), metronidazole (100 mg/kg), and vancomycin (50 mg/kg) in 200 μl of sterile water for a week. This was supplemented with *ad libitum* access to ampicillin (1 mg/ml) in their drinking water.

### Construction of colon or DRG Piezo2 knockdown mice

We utilized adeno-associated virus-9 (AAV9) vectors carrying Piezo2 shRNA (rAAV-U6-shRNA (Piezo2-sh1)-U6-shRNA (Piezo2-sh2)-U6-shRNA (Piezo2-sh3)-U6-shRNA (Piezo2-sh4)-CMV-EGFP-pA) and control-shRNA (rAAV-U6-shRNA (scramble)-U6-shRNA (scramble)-U6-shRNA (scramble)-U6-shRNA (scramble)-CMV-EGFP-pA), which were constructed by Wuhan BrainVTA. The silencing sequences targeting Piezo2 genes followed a previous study^21^ and are listed in Supplementary Table S2.

To create colon or DRG Piezo2 knockdown (KD) mice, AAV9 was administered via intraperitoneal or intrathecal injection at a dose of 1  ×  10^12^ viral genome particles per mouse. Adequate transfection was observed three weeks or three days after AAV9 administration, respectively, preceding Abx treatment and FMT.

### pGF mice receive FMT from IBS-D patients or HC

Prior to FMT, all pGF mice underwent a 4-day antibiotic-free period to eliminate any potential influence of antibiotics on the transplanted microbiota.

Five donors from each group of HC and IBS-D patients, matched for gender, age, and BMI, were selected for FMT (donor details are provided in Supplementary Table S3). In brief, 200 mg of frozen stool samples from five donors in each group were pooled and suspended in 10 mL of sterile oxygen-free phosphate-buffered saline (PBS) containing 20% (v/v) glycerol within a sterile anaerobic chamber. The suspension was then aliquoted into four portions and stored at  − 80°C. Prior to each use, an aliquot was thawed to avoid repeated freezing and thawing.

The pGF mice were randomly assigned to two groups: FMT-control (*n* = 23) and FMT-IBS (*n* = 23). They were colonized via oral gavage with 200 μl of the suspension from HC or IBS-D patients, respectively, twice weekly for two weeks. After each gavage, 1 mL of the suspension was splashed onto the bedding and the inner wall of each mouse cage.^22^

### Co-housing experiments

Given the coprophagous habits of mice, microbiota can be horizontally transmitted between them, leading to homogenization of the microbiota among mice housed in the same cage and resulting in similar gut microbiota composition. Therefore, we adopted and modified the methods from previous studies.^23–25^ The experimental procedure for co-housing is as follows. A totally of 24 mice were randomly divided into two groups. Six mice were housed in a single cage. One mouse from each cage was randomly selected to receive oral gavage FMT (sourced from either HC or IBS-D patients) twice weekly for two consecutive weeks. Following gavage, the mice were returned to their original cages (see Supplementary Figure S1). In the subsequent two weeks, the remaining five mice in each cage, which did not receive FMT gavage, will acquire the gut microbiota from either IBS-D patients (cFMT-IBS group) or HC (cFMT-control group) through coprophagy.

### Cultures and media of anaerobic bacteria

The strains of *Akkermansia muciniphila* and *Fusobacterium varium* utilized in this study were obtained from the State Key Laboratory of Microbial Resources at the Institute of Microbiology, Chinese Academy of Sciences, Beijing, P.R. China. These bacterial strains were cultivated anaerobically in a modified version of commercial Modified Gifu Anaerobic Medium (MGAM) at 37°C until required for use.

### *Intervention experiment with Akkermansia muciniphila and* Fusobacterium varium

*Akkermansia muciniphila* (*A. muciniphila*) and *Fusobacterium varium* (*F. varium*) were inoculated and incubated at 37°C for 7 and 3 days, respectively, to achieve the stationary phase. The colony-forming units (CFU) of both cultures were ascertained by plating series dilutions on modified MGAM agar plates and subsequently counting the colonies. The bacteria were harvested from the cultures through centrifugation at 8,228 g for 15 min within an anaerobic chamber. They were then washed twice and re-suspended in oxygen-free PBS containing 20% (v/v) glycerol to attain a final concentration of 2  ×  10^9^ CFU per 200 μl.

Prior to administrating the bacteria, the pGF mice undergoing Abx treatment were provided a 4-day antibiotic-free period to eliminate any residual antibiotic effects on the anaerobic bacteria.

Subsequently, the pGF mice were randomly assigned to either the control group (vehicle) or the experimental groups (*A. muciniphila* group or *F. varium* group), with *n* = 10 for each group. The experimental groups received oral gavage of 200 μl of *A. muciniphila* or *F. varium* suspension every other day for two weeks, respectively, alone with bedding inoculation.

### Intestinal motility testing

The intestinal motility of the subjects was assessed following an experimental protocol routinely employed in our laboratory,^26,27^ which is succinctly outlined below.

### Colonic transit time

The colonic transit speed of the mice was evaluated using the bead expulsion test.^28^ In brief, the mice were fasted for 12 hours, with water available. Subsequently, they were lightly anesthetized with isoflurane (RWD, Shenzhen, China), and a 2.5 mm spherical plastic bead coated with glycerol was carefully inserted into the distal colon using a glass rod, placing it 2 centimeters from the anus. The mice were then individually placed in cages without bedding, and the time from bead insertion to expulsion was recorded. This experiment was repeated twice, with a 6-hour interval between trials. The bead expulsion latency was determined by calculating the average of these two measurements.

### Whole-intestinal transit time

Each mouse received 200 μl of carmine red solution (composed of 6% carmine powder dissolved in a 0.5% carboxymethylcellulose aqueous solution) via oral gavage. The time until the first stool containing carmine was discharged was recorded, representing the whole-intestinal transit time.

### Fecal pellet output and fecal water content

The mice were individually placed in dry, bedding-free cages for a duration of one hour. Fecal pellets were collected at 15-minutes intervals during this period and immediately covered with centrifuge tube caps to prevent evaporation. After 1 hour, the collected fecal pellets were counted and weighed, yielding the wet weight. Subsequently, the fecal pellets were dried in a drying oven at 60°C for 24 hours and then reweighed to obtain the dry weight. The stool water content was then calculated using the wet weight and dry weight to determine the proportion of water in the fecal pellets.

### Colorectal distension-electromyogram (CRD-EMG)

The CRD-EMG procedure was conducted based on established methods with certain modifications.^29^ A latex balloon, measuring 1 cm in width, and 1.5 cm in length, was attached to a thin catheter (1 mm inner diameter, 1.5 mm outer diameter) at its tip, serving as a pressure-sensitive balloon. Prior to the visceral sensitivity testing, the mice were fasted for 24 hours with water accessible. Following light anesthesia induced by isoflurane, a glycerol-coated balloon was carefully inserted into the mouse’s rectum, positioning its distal end approximately 1 cm from the anus. The catheter attached to the balloon was securely fastened to the base of the mouse’s tail using adhesive tape. The distal end of the balloon catheter was then connected to a Distender Series IIR (G&J Electronics, Ontario, Canada) and a syringe through a three-way stopcock. After a 20-minute adaptation period, the balloon was swiftly inflated to pressures of 20 mmHg, 40 mmHg, and 60 mmHg in a randomized sequence. Each inflation was maintained for 20 seconds, with a 4-minute interval between inflations. This process was repeated three times for each pressure level to ensure accurate measurements. The signals were collected and analyzed using the Taimen Biological Signal Acquisition System software (Chengdu Taimen Software Co. LTD, Chengdu, China). Changes in visceral sensation were quantified by subtracting the area under the curve (AUC) during the 20- seconds pre-distension baseline from the AUC recorded during the 20 seconds of distension.

### Enzyme-linked immunosorbent assay (ELISA)

The level of 5-HT was assessed in both mouse serum and distal colon using an enzyme-linked immunosorbent assay kit (CEA808Ge, Cloud Clone, Wuhan, China). Briefly, under anesthesia, serum was collected by eyeball extraction and centrifuged at 4000 rpm for 10 min at 4°C. Following serum collection, the mice were immediately euthanized via cervical dislocation. The distal colon tissue was then homogenized in ice-cold PBS (0.01 M, pH = 7.4). After centrifugation at 3000 rpm for 20 min at 4°C, the supernatant was collected. The concentration of 5-HT was quantified using the ELISA kit according to the manufacturer’s instructions. Absorbance was measured at 450 nm using an 800^TM^ TS fully automatic microplate reader (Biotek, U.S.).

### Histology and electron microscopy

Hematoxylin-eosin (H&E) staining was used to evaluate the colon mucosa of the mouse. Following anesthesia and cervical dislocation, colon samples were fixed in a 10% formaldehyde-PBS solution for 24 hours, dehydrated, embedded in paraffin, and sectioned into 5 μm-thick slices. After deparaffinization and hydration, the sections were stained with eosin and hematoxylin, mounted with neutral gum, and immediately imaged using a NanoZoomer S210 (Tokyo, Japan).

### Immunofluorescence staining

Mice were deeply anesthetized with sodium pentobarbital (100 mg/kg) and perfused with preheated (37°C) saline from the ascending aorta followed by cold (4°C) 4% paraformaldehyde in 0.1 M phosphate-buffered saline (PBS, pH 7.4). The T12-L1 and L6-S1 DRGs and distal colon were rapidly removed, post-fixed in 4% paraformaldehyde for 6 h, and cryoprotected in a 30% sucrose solution overnight at 4°C. Subsequently, the tissue was embedded in OCT compound and stored at − 80°C. The tissues were then sectioned into 8 μm slices using a cryostat, mounted on gelatin-coated glass slides, and stored at  − 80°C until immunofluorescence staining was performed.

Following blocking with a solution of 5% normal donkey serum and 0.25% Triton X-100 in PBS for 20 min at 37°C, the sections were incubated overnight at 4°C with primary antibodies diluted in PBS containing the same concentrations of normal donkey serum and Triton X-100. After thorough washing, the sections were subsequently incubated with secondary antibody for 2 h at room temperature. Next, nuclear staining was performed using Hochest 33,342 (1:2000; #c3001, Solarbio, Beijing, China) for 10 min at room temperature. Finally, the sections were washed with PBS and examined under a confocal microscope (Leica TCS-SP8 DIVE, Germany). The antibodies used and their respective concentrations are detailed in Supplementary Table S5.

### Western blot analysis

After anesthesia and cervical dislocation, the T12-L1 and L6-S1 DRG and a 1 cm segment of the distal colon were dissected and immediately frozen at − 80°C. Total protein was extracted using RIPA lysis buffer containing protease inhibitor and phenylmethylsulfonylfluoride (PMSF) (#P6730, Solarbio, China), alone with protein phosphatase inhibitor (#P1260, Solarbio, China). After complete lysis, the lysates were centrifuged at 12,000 rpm for 10 min at 4°C, and the supernatant was collected. Protein concentration was quantified using a Pierce^TM^ BCA Protein Assay kit (Cat#23225, Thermo Scientific, Waltham, MA, USA).

A protein lysate containing 50 μg of protein was separated by Bis-Tris 4%-20% sodium dodecyl sulfate-polyacrylamide gel electrophoresis (SDS-PAGE) and transferred to polyvinylidene fluoride (PVDF) membranes. The membranes were blocked with 25 ml of blocking buffer (5% nonfat dry milk in Tris-buffered saline) containing 0.1% Tween (TBST) for 1 h at room temperature, following by overnight incubation with the primary antibodies at 4°C. After three washes with TBST buffer, the membranes were incubated with the corresponding secondary antibodies for 1 h. Protein band intensities were detected using Immobilon Western Chemiluminescent HRP substrate (#WBKLS0500, Millipore, Darmstadt, Germany). The optical densities of the immunoreactive bands were quantified using ImageJ software (National Institutes of Health, Bethesda, MD). The antibodies used and their concentrations are listed in Supplementary Table S5.

### Gut microbiota collection and analysis

Genomic DNA was extracted from the fecal microbial community of 100–200 mg human stool sample or 50–100 mg mouse fecal sample using the E.Z.N.A.® soil DNA Kit (Omega Bio-tek, Norcross, GA, U.S.) following the manufacturer’s instructions. Sterile water blank samples served as controls. Subsequently, the extracted genomic DNA was visualized on a 1% agarose gel, and its concentration and purity were determined using a NanoDrop 2000 UV-vis spectrophotometer (Thermo Scientific, Wilmington, USA). The hypervariable V3-V4 region of the bacterial 16S rRNA gene was amplified with the primer pairs 338F (5'-ACTCCTACGGGAGGCAGCAG-3') and 806R (5'-GGACTACHVGGGTWTCTAAT-3') on an ABI GeneAmp® 9700 PCR Thermocycler (ABI, CA, USA). The PCR products were then extracted from a 2% agarose gel, purified with the AxyPrep DNA Gel Extraction Kit (Axygen Biosciences, Union City, CA, USA), and quantified using a Quantus™ Fluorometer (Promega, USA). The purified amplicons were pooled in equimolar amounts and subjected to paired-end sequencing on an Illumina MiSeq PE300 platform (Illumina, San Diego, CA, U.S.) using standard protocols provided by Majorbio Bio-Pharm Technology Co. Ltd. (Shanghai, China).

The resulting microbiome samples were analyzed utilizing the Parallel-Meta Suite^30^ alongside the SILVA database (version 138, https://www.arb-silva.de/.)^31^ with an operational taxonomic unit similarity threshold set at 97%. Functional profiles were predicted using the Phylogenetic Investigation of Communities by Reconstruction of Unobserved States (PICRUSt2).^32^ Alpha diversity was evaluated by calculating the Shannon, Simpson, and Chao1 indices for each sample, and the results were illustrated using boxplots. Differences in alpha diversity indices among groups were compared using the Wilcoxon test. For beta diversity analysis, principal component analysis (PCoA), partial least-square discriminant analysis (PLS-DA) and PERMANOVA tests were conducted, employing the meta-storm distance algorithm.^33^ The linear discriminant analysis (LDA) effect size (LEfSe) method was utilized to identify microbial taxa that significantly differed between groups, with a threshold LDA score set at >2. Additionally, co-occurrence network analysis was performed using Cytoscape,^34^ considering statistical significance at *p* < 0.05 and a correlation coefficient threshold of | r | ≥ 0.5. Heatmap correlation analysis was performed using Spearman’s method.

All amplicon sequencing data have been submitted to the National Genomics Data Center (NGDC) and are accessible under BioProject accession number PRJCA035313 and PRJCA035312.

### Untargeted metabolism analysis

Sample preparation: After anesthesia and cervical dislocation, the cecum was weighed, freeze-dried, and ground in a grinder at 65 hz for 1 min. Metabolites were extracted using a pre-cooled methanol-acetonitrile-water mixture, and then subjected to ultrasonic agitation in an ice bath for 1 hour. After centrifugated at 4°C and 14,000 g for 20 min, the supernatant was concentrated.

Ultra-High Performance Liquid Chromatography (UHPLC)-Mass Spectrometry (MS)/MS analysis: Metabolomics profiling was analyzed using a UPLC-ESI-Q-Orbitrap-MS system (UHPLC, Shimadzu Nexera X2 LC-30AD， Shimadzu，Japan) coupled with a Q-Exactive Plus (Thermo Scientific, San Jose, USA).

For liquid chromatography (LC) separation, an ACQUITY UPLC® HSS T3 column (2.1  ×  100 mm, 1.8 μm) (Waters, Milford, MA, USA) was employed. Both positive-mode and negative-mode electrospray ionization (ESI) were applied for MS data acquisition.

Data preprocessing and filtering: Raw MS data was processed using MS-DIAL. Metabolites were identified based on accurate mass (mass tolerance of <10 ppm) and MS/MS data (mass tolerance < 0.02 Da) matched against public databases (such as HMDB) and an internal library. Only features with > 50% non-zero values in at least one group were retained.

Multivariate statistical analysis: All multivariate data analyses and modeling were performed using R (version: 4.0.3) and R packages. Data were mean-centered using Pareto scaling. Models were constructed using orthogonal partial least-square discriminant analysis (OPLS-DA). All models were valuated using permutation tests.

Discriminating metabolites were identified based on OPLS-DA model variable influence on projection (VIP) values >1 and a two-tailed t-test with *p* < 0.05. Fold change was calculated as the logarithm of the average mass response ratio. Clustering analysis was performed on the differential metabolites.

For more detailed information on the experimental conditions for untargeted metabolite analysis, please refer to previous studies.^35^

The untargeted metabolism data have been deposited in the NGDC and can be found under BioProject accession number PRJCA035357.

### Molecular docking

The high resolution Cryo-EM structure of the mammalian tactile channel Piezo2 (PDB ID: 6KG7)^36^ was retrieved from the Protein Data Bank (PDB) database. Subsequent preparation was carried out using the Prepare Protein and Minimization protocols in Discovery Studio 2020 (DS; BIOVIA-Dassault Systèmes, San Diego, USA). Leveraging an artificial intelligence algorithm, the pharmacophore generation protocol from Drug Target Space (DTS; EGR Health, Beijing, China) was applied to identify the most representative features of the Piezo2 interface.

### Statistical analysis

Data are presented as mean ± SEM or median (interquartile range [IQR]). The Shapiro-Wilk and Kolmogorov-Smirnov tests were employed to assess the normal distribution of the data. Comparisons between two groups were conducted using Student’s t-test or non-parametric tests (Mann-Whitney test). For comparison involving more than two groups, a one-way analysis of variance (ANOVA) was performed, followed by Tukey’s multiple comparison test. In cases where two variables were involved, a two-way ANOVA was applied, followed by either Tukey’s or Sidak’s multiple comparison test. Correlations between indicators were analyzed using the Spearman test, and a correlation heat map was created using ChiPlot (https://www.chiplot.online/). The specific statistical tests employed for each panel are detailed in the figure legends. Statistical significance was defined as a *p* value < 0.05. All statistical analysis were conducted using GraphPad Prism 10 software (GraphPad Software, CA, USA).

The flowchart outlining the entire study is provided in Supplementary Figure S2.

## Results

### Piezo2 expression in the colon correlates with symptoms in patients with IBS-D

**Figure 1.**
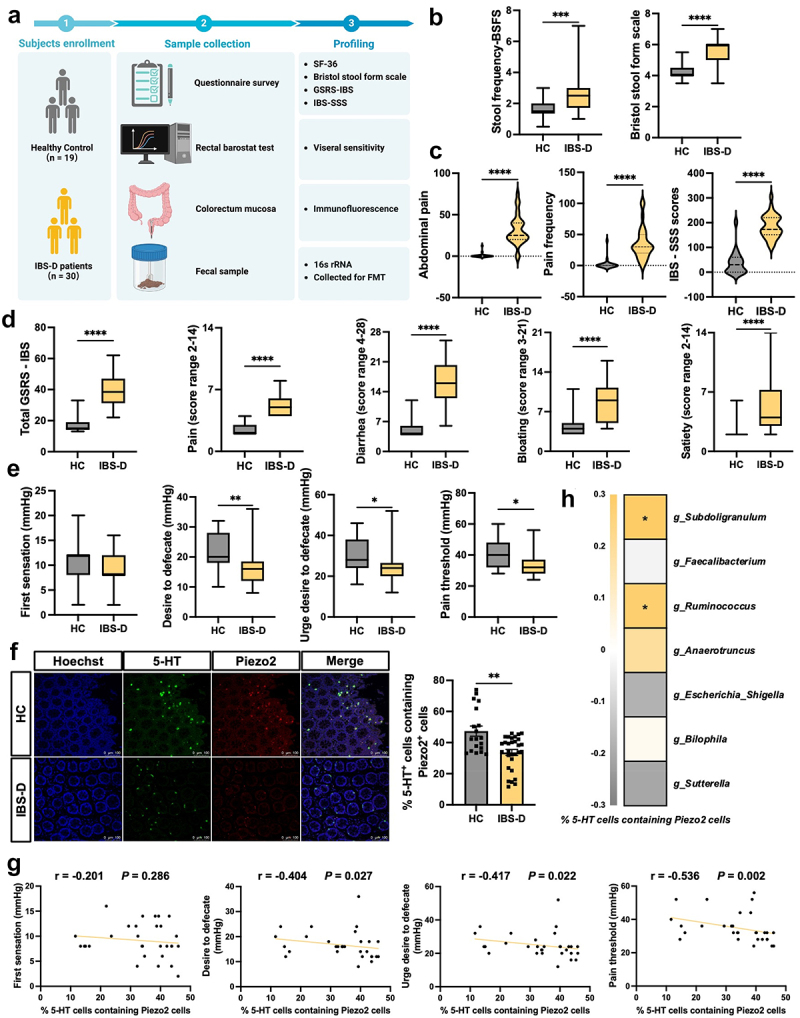
Decreased Piezo2^+^ 5-HT^+^ cells in IBS-D patients with visceral hypersensitivity and gut dysbiosis.

Subjects who completed the questionnaire, underwent barostat testing, as well as colonoscopy with sigmoid mucosal biopsy were included in the study. Ultimately, 19 HC and 30 patients diagnosed with IBS-D were enrolled. The two groups were age- and BMI-matched. The experimental research content and sample collection process are illustrated in Figure 1(a). Our results demonstrated that IBS-D patients exhibited significantly lower scores in the Physical Component Summary (PCS), Mental Component Summary (MCS), and sub-scores of the SF-36 questionnaire compared to HC (Supplementary Figure S3A-B). Additionally, their BSFS score and stool frequency-BSFS score were notably higher (Figure 1(b)). Furthermore, abdominal pain intensity, pain frequency and the total score of the IBS-SSS were all significantly elevated in the IBS-D group (Figure 1(c)). Similarly, the total score and all sub-scores of the GSRS were also increased in these patients (Figure 1(d)). Rectal barostat studies revealed that the threshold of first sensation, urge to defecate, and pain were significantly reduced in IBS-D patients compared to HC (Figure 1(e)). Analysis of fecal 16S rRNA uncovered substantial differences in gut microbiota composition between the groups, evident in variations of α- and β-diversity (Supplementary Figure S3C-D). The p-value for PERMANOVA tests on gut microbiota β-diversity between HC and IBS-D patients was 0.328, which was not statistically significant. However, PLS-DA results showed a clear distinction between the two groups at the genus level, with a p-value of 0.09 (Supplementary Figure S3D). Furthermore, there were distinct differences in gut microbiota composition at both the phylum and genus levels (Supplementary Figure S3E-F). Specifically, genera such as *g_Sutterella*, *g_Bilophila* and *g_Escherichia_Shigella* were more abundant in IBS-D patients, while *g_Subdoligranulum*, *g_Faecalibacterium*, *g_Rumminococcus* and *g_Anaerostipes* were more prevalent in the HC group (Supplementary Figure S3G).

(a) Overview of participates recruitment and sample collection protocol. (b) Results from the BSFS questionnaire. (c) Results from the IBS-SSS questionnaire. (d) Results from the GSRS questionnaire. (e) Results of the rectal barostat test. (f) Immunofluorescent staining for Piezo2 and 5-HT were performed on sigmoid colonic mucosa sections. (g) Correlation analysis between Piezo2^+^ 5-HT^+^cells and visceral sensitivity. (h) Correlation analysis between Piezo2^+^ 5-HT^+^cells and differential expression of gut microbiota. Data are presented as median ± IQR. HC, *n* = 19; IBS-D, *n* = 30. Nonparametric test with Mann-Whitney test and spearman correlation analysis. *p < 0.05, **p < 0.01, ***p < 0.001, ****p < 0.0001.

To investigate the expression of Piezo2, we conducted immunofluorescence staining on sigmoid colon mucosa samples obtained through colonoscopic biopsies. Our results showed that a significantly lower proportion of Piezo2^+^ cells within the 5-HT^+^ cells population in IBS-D patients (Figure 1(f)).

A more in-depth correlation analysis indicated that the proportion of Piezo2^+^ 5-HT^+^ cells was negatively correlated with the pain threshold as determined by rectal barostat studies in IBS-D patients (Figure 1(g)). Conversely, it showed a significant positive correlation with the abundance of *g_Subdoligranulum* and *g_Rumminococcus* (Figure 1(h)).

Briefly, IBS-D patients exhibit a markedly decreased ratio of 5-HT^+^ cells expressing Piezo2^+^, which was positively correlated with their visceral hypersensitivity and associated with alterations in the gut microbiota.

### The FMT derived from IBS-D patients induced visceral hypersensitivity and gut dysmotility in pGF mice

To explore whether Piezo2 is regulated by the gut microbiota, subsequently influencing visceral sensitivity and intestinal motility, we conducted the following study. Initially, we selected gender-, age-, and BMI-matched subjects from both the HC and IBS-D groups to serve as donors (Supplementary Table S3). Subsequently, we performed a FMT experiment transferring microbiota from humans to mice. We proceeded to analyze the gut microbiota presented in the donor’s feces samples (Supplementary Figure S4). The results showed a statistically significant difference in β-diversity between the HC group donors (D_CTRL) and the IBS-D group donors (D_IBS), with a PERMANOVA test yielding a *p* value of 0.02. However, no significant difference was observed in α-diversity was (Supplementary Figure S4A). PCoA further demonstrated a clear distinction between the two groups, highlighting notable disparities in their gut microbiota composition (Supplementary Figure S4B). The Venn diagram provided additional insight into the distinct gut microbiota profiles of the two donor groups (Supplementary Figure S4C). Furthermore, significant differences in gut microbiota composition were evident at both the phylum and genus levels (Supplementary Figure S4D-E). Specifically, *g_Escherichia_Shigella* was more abundant in the D_IBS group, whereas *g_Ruminococcaceae_Group* was more prevalent in the D_CTRL group (Supplementary Figure S4F).

**Figure 2.**
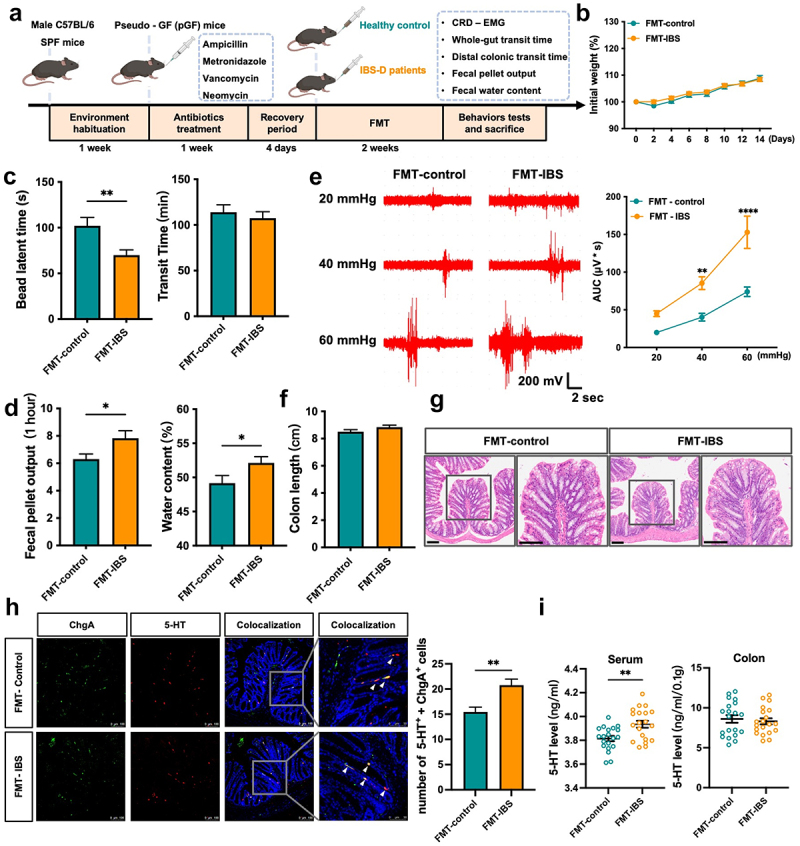
FMT from IBS-D patients induced visceral hypersensitivity and gut dysmotility in pGF mice.

We established a pGF mouse model by depleting intestinal microbiota using a cocktail of four antibiotics administered to SPF mice for one week, followed by a four-day recovery period. Then, we conducted FMT twice weekly for two weeks, using donors from both HC (FMT-control) and patients with IBS-D (FMT-IBS), to these pGF mice (Figure 2(a)). During the FMT procedure, there were no significant differences in body weight between the two groups (Figure 2(b)). Compared to the FMT-control group, mice in the FMT-IBS group showed a shorter colon transit time, higher fecal pellets output, and greater fecal water content. However, there was no difference in whole-intestinal transit time between the two groups (Figure 2(c-d)). Additionally, the FMT-IBS group demonstrated significantly increased visceral sensitivity at 40 mmHg and 60 mmHg pressure points in the CRD- EMG test (Figure 2(e)).

(a) Schematic diagram of the IBS model constructed by FMT in pGF mice. (b) Body weight of the mice; *n* = 23 mice per group; using two-way ANOVA with Sidak’s post-hoc test. (c) Colon transit time (left) and whole-intestinal transit time (right); *n* = 23 mice per group; using unpaired t-test. (d) Number of fecal pellet outputs (left) and water content of fecal pellet (right); *n* = 23 mice per group; using unpaired t-test. (e) Results of the CRD-EMG test; *n* = 11 mice per group; using two-way ANOVA with Sidak’s post-hoc test. (f) Colon length of pGF mice after FMT; *n* = 23 mice per group; using unpaired t-test. (g) H&E staining of the distal colon of pGF mice after FMT. (h) Quantification of 5-HT^+^ ECs cells in the distal colon; *n* = 12 mice per group, using the nonparametric test with the Mann-Whitney test. (i) Levels of 5-HT in serum and colon; *n* = 20 mice per group; both analyzed using the nonparametric test with the Mann-Whitney test. Data are presented as mean ± SEM. Scale bar = 100 μm. *p < 0.05, **p < 0.01, ****p < 0.0001.

Furthermore, there were no notable differences in colon length between the groups (Figure 2(f)), and H&E staining revealed no overt tissue damage or inflammatory cell infiltration in the colon of either group (Figure 2(g)).

5-HT, a crucial neurotransmitter implicated in the pathophysiology of IBS, is primarily synthesized by enterochromaffin cells (ECs). Therefore, we quantified ECs in the colon and found an increase in 5-HT^+^ ChgA^+^ cells in the FMT-IBS group (Figure 2(h)). Subsequent enzyme-linked immunosorbent assay (ELISA) analysis confirmed elevated 5-HT levels in the serum, but not in the colon, of the FMT-IBS group (Figure 2(i)).

### Piezo2 was upregulated in the colon and DRG of pGF mice following FMT from IBS-D patients

**Figure 3.**
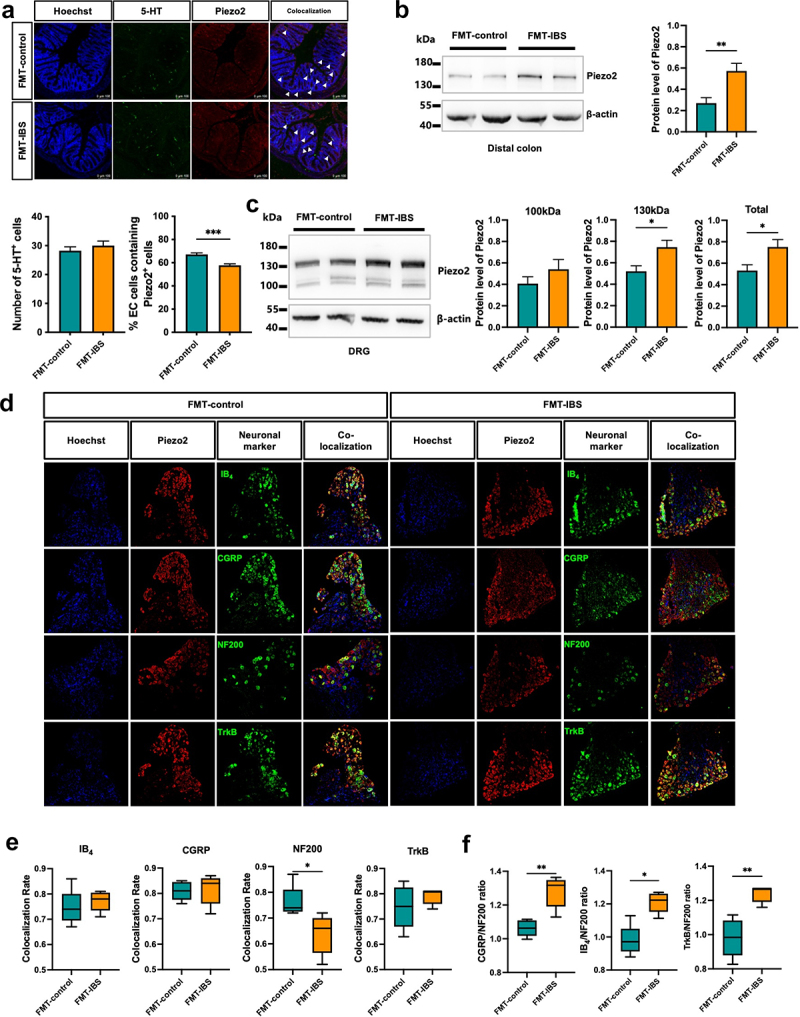
Elevated Piezo2 levels were in the colon and DRG of pGF mice following FMT from IBS-D patients.

Piezo2 predominantly expressed in ECs, plays a crucial role in the release of the vital neurotransmitter 5-HT.^8,9^ We investigated Piezo2 expression in the colon of pGF mice after FMT. Immunofluorescence staining revealed no significant difference in the number of 5-HT^+^ cells between the two groups (Figure 3(a)). However, the FMT-IBS group showed a reduction in the percentage of Piezo2^+^ cells among 5-HT^+^ cells in the distal colon mucosa (Figure 3(a)), aligning with findings in the sigmoid colon mucosa of IBS-D patients. Notably, western blot analysis demonstrated an elevation in Piezo2 protein levels in the distal colon of the FMT-IBS group (Figure 3(b)).

(a). Immunofluorescence staining of Piezo2 and 5-HT in the distal colon of pGF mice after FMT; *n* = 12 mice per group using a nonparametric test with the Mann-Whitney test. (b) Protein level of Piezo2 in the distal colon; FMT-control: *n* = 10, FMT-IBS: *n* = 9. (c) Protein level of Piezo2 in the DRG; FMT-control: *n* = 10, FMT-IBS: *n* = 11. (d) Immunofluorescence staining of IB_4_, CGRP, NF200, TrkB and Piezo2 in the L6 DRG of pGF mice after FMT; *n* = 5 mice per group; using the nonparametric test with the Mann-Whitney test. (e) Colocalization rate of Piezo2 with various neuronal markers; *n* = 5 mice per group; using the nonparametric test with the Mann-Whitney test. (f) Colocalization rate ratio of pain-related markers to NF200; *n* = 5 mice per group; using the nonparametric test with the Mann-Whitney test. Data are presented as mean ± SEM or median ± IQR. *p < 0.05, **p < 0.01, ***p < 0.001.

Furthermore, western blot results showed a significant increase in Piezo2 protein levels in T12-L1 and L6-S1 DRG neurons, which innervate the colon (Figure 3(c)). Immunofluorescence staining of L6 DRG for Piezo2 and various neuronal markers revealed that Piezo2 is expressed across DRG neurons of different diameters. In the FMT-IBS group, the colocalization rate of neurofilament 200 (NF200^+^) and Piezo2^+^ neurons decreased, while the other three pain-related markers showed a non-significant increasing trend (Figure 3(d,e)). To further analysis the colocalization rate ratios, we found that the ratios of isolectin B4 (IB_4_)/NF200, calcitonin gene related peptide (CGRP)/NF200 and tropomyosin receptor kinase B (TrkB)/NF200 were significantly higher in the FMT-IBS group compared to the FMT-control group (Figure 3(f)), indicating that more pain-related neurons colocalized with Piezo2.

### Co-housing with FMT-IBS pGF mice also induces gut dysfunction and upregulation of Piezo2

Considering the pivotal role of central stress in the development of IBS, we employed a co-housing method to facilitate microbiota transfer between mice, assigning them to either the FMT-control or FMT-IBS (designated as cFMT-control and cFMT-IBS, respectively). This approach eliminated the stress associated with direct fecal microbiota gavage by leveraging the natural coprophagic behavior of mice (Supplementary Figure S1). The results showed no significant difference in body weight between the two groups (Supplementary Figure S5A). However, the cFMT-IBS group demonstrated a shortened colon transit time, an increase in fecal pellets output, and higher fecal water content, while the whole-intestinal transit time remained unaffected (Supplementary Figure S5B). Notably, the visceral sensitivity of the cFMT-IBS group was significantly elevated (Supplementary Figure S5C). H&E staining of colon tissues revealed no obvious inflammation in either group following FMT (Supplementary Figure S5D). Subsequent analysis using western blot detected an increase of Piezo2 levels in both colon tissue and DRG neurons (Supplementary Figure S5E-F).

### Piezo2 plays a role in the development of visceral hypersensitivity and gut dysmotility induced by FMT from IBS-D patients

**Figure 4.**
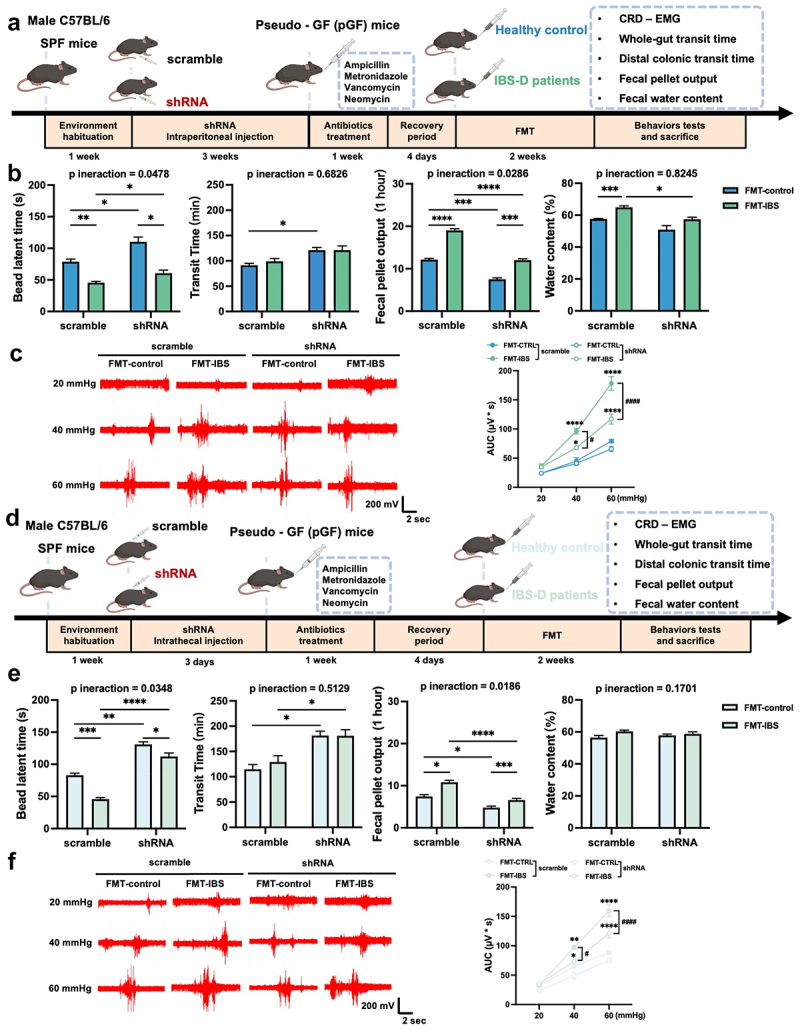
Piezo2 knockdown in the colon or DRG partially alleviate visceral hypersensitivity and gut dysmotility induced by FMT.

Piezo2 has been implicated in the development of visceral hypersensitivity and gut dysmotility observed in mice subjected to FMT from patients with IBS-D. To elucidate the specific role of Piezo2 in this process, we generated Piezo2 KD pGF mice in both the colon (Figure 4(a)) and DRG (Figure 4(d)).

(a) Schematic diagram of the IBS model constructed through FMT in colon Piezo2 KD pGF mice. (b) Assessments include colon transit time, whole-intestinal transit time, fecal pellet output count, and fecal pellet water content. *n* = 8 mice per group, using two-way ANOVA with Tukey’s post-hoc test. (c) CRD-EMG results. *n* = 8 mice per group; using three-way ANOVA with Tukey’s post-hoc test. (d) Schematic diagram of the IBS model created via FMT in DRG Piezo2 KD pGF mice. (e) Measurements encompass colon transit time, whole-intestinal transit time, fecal pellet output, and fecal pellet water content. *n* = 7–10 mice per group; using two-way ANOVA with Tukey’s post-hoc test. (f) CRD-EMG results. *n* = 7–10 mice per group; using three-way ANOVA with Tukey’s post-hoc test. Data are presented as mean ± SEM. *p < 0.05, **p < 0.01, ***p < 0.001, ****p < 0.0001.

Initially, we administered Piezo2 shRNA via intraperitoneal injection, effectively downregulating Piezo2 expression in the colon without eliciting notable colonic inflammation (Supplementary Figure S6A-B). During the FMT procedure, body weight remained stable across all four experimental groups (Supplementary Figure S6C), and no evident inflammation was observed in the colon post-FMT (Supplementary Figure S6D). Mice with colonic Piezo2 KD exhibited an extended colonic transit time and a decrease in fecal pellets output. However, no substantial differences were noted in whole-intestinal transit time or fecal water content (Figure 4(b)). Furthermore, visceral hypersensitivity was partially ameliorated in these mice (Figure 4(c)). Likewise, intrathecal injection of Piezo2 shRNA successfully downregulated Piezo2 expression in the DRG (Supplementary Figure S6E-F). No significant disparities in body weight or colonic inflammation were detected among the four groups after FMT (Supplementary Figure S6G-H). DRG Piezo2 KD mice also showed improvements in shortened colon transit time, increased fecal pellets output, and heightened visceral sensitivity, yet no changes were observed in whole-intestinal transit time or fecal water content (Figure 4(e,f)).

### Microbiome analysis reveals that Fusobacterium varium may play a pivotal role in regulating Piezo2

**Figure 5.**
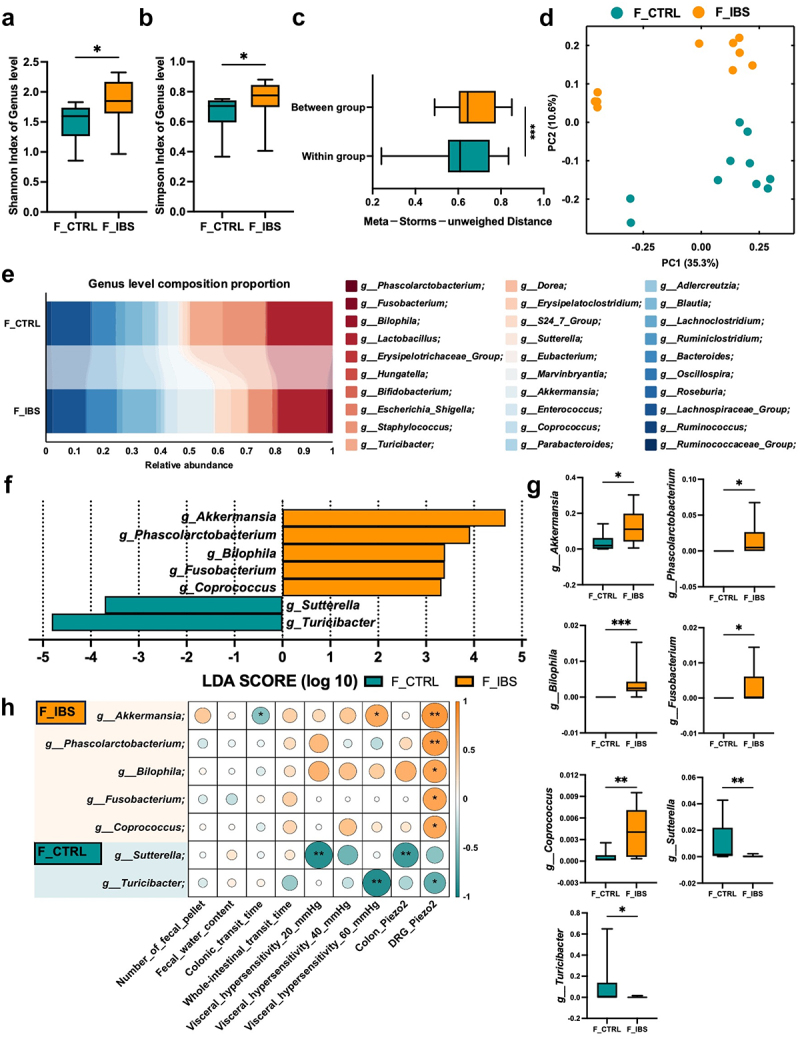
The 16S rRNA of pGF mice after FMT from IBS-D patients.

We conducted 16S rRNA sequencing on fecal samples from mice following FMT. The results showed notable differences in both α- and β-diversity between the two groups (Figure 5(a–c)). PCoA further highlighted distinct compositions of gut microbiota between these groups (Figure 5(d)). Notably, differences were evident at both the phylum (Supplementary Figure S7A) and genus (Figure 5(e)) levels. A supervised random forest analysis identified p_Fusobacteria and *g_Bilophila* as the primary contributors to the classification of microbiota status (Supplementary Figure S7B and S8).

(a–b) α-diversity (Shannon index and Simpson index) at the genus level. (c) β-diversity at the genus level. (d) PCoA results. (e) Genus level composition of the two groups of mice. (f-g) Difference abundance of genus between the two groups. (h) Correlation analysis between differentially abundance genus and GI symptoms of pGF mice after FMT, as well as Piezo2 protein levels. Data are presented as median ± IQR. *n* = 10 mice per group. Nonparametric test with Mann-Whitney test. *p < 0.05, **p < 0.01, ***p < 0.001.

At the genus level, we identified seven gut microbiota with differential expressed. Among them, *g_Turicibacter* and *g_Sutterella* were enriched in the FMT-control group, whereas *g_Akkermansia*, *g_Phascolarctobacterium*, *g_Bilophila*, *g_Fusobacterium* and *g_Coprococcus* were more abundant in the FMT-IBS group (Figure 5(f-g)). Correlation analysis further showed that Piezo2 expression in the DRG was positively correlated with the five bacteria enriched in the FMT-IBS group. Visceral sensitivity exhibited a significant negative correlation with *g_Sutterella* and *g_Turicibacter*, and a positive correlation with *g_Akkermansia*. Notably, *g_Akkermansia* demonstrated a negative correlation with colonic transit time (Figure 5(h)).

We further compared the feces of donors and mice after FMT. The results revealed a significant difference in α-diversity between D_CTRL (representing donors from the HC group) and F_CTRL (representing mice receiving FMT from HC donors), but not between D_IBS (representing donors from the IBS-D group) and F_IBS (representing mice receiving FMT from IBS-D patients) (Supplementary Figure S9A). However, the β-diversity was significantly different, with a PERMANOVA test yielding a p-value of 0.001. It was observed that D_CTRL was closer to D_IBS, and F_CTRL was closer to F_IBS (Supplementary Figure S9B-C). Notable differences in gut microbiota composition were observed at both the phylum (Supplementary Figure S9D) and genus (Supplementary Figure S9E) levels. LEfSe analysis revealed bacteria that were enriched in different groups at the genus level (Supplementary Figure S9F).

Furthermore, we compared the distances between donors and mice, finding that the between-group distance was significantly greater than the within-group distance. This is consistent with the PCoA plot and indirectly suggests the presence of bacterial colonization “screening” during FMT (Supplementary Figure S9G). Additionally, the distance between D_IBS and F_IBS was significantly smaller than that between D_CTRL and F_CTRL (Supplementary Figure S9H), indicating that the gut microbiota structure of mice received FMT from IBS-D patients was more similar to that of the IBS-D patients themselves.

Based on our results and with reference to the literatures,^37–41^ we focused on two species from the enriched genera in the FMT-IBS group, namely *A. muciniphila* and *F. varium*, for subsequent experiments. pGF mice were colonized with one of these two species every other day for 2 weeks (Supplementary Figure S9A). Neither species significantly affected body weight nor induced colon inflammation (Supplementary Figure S10B, E, F, I). However, *A. muciniphila* prolonged colon transit time and reduced fecal pellet output in pGF mice, without affecting whole-intestinal transit time or fecal water content (Supplementary Figure S10C). Additionally, it alleviated increased the increase in visceral sensitivity (Supplementary Figure S10D). In contrast, *F. varium* significantly shortened colonic transit time, increased fecal pellets output, and elevated fecal water content in pGF mice, while having no effect on whole-intestinal transit time (Supplementary Figure S10G). Notably, visceral sensitivity was significantly increased (Supplementary Figure S10H).

To investigate the potential role of *F. varium* in regulating Piezo2 to mediate visceral hypersensitivity and colonic dysmotility, we conducted western blot analysis on mice that had been exposed to *F. varium*. Notably, we observed a significantly increase in the level of Piezo2 protein in both the colon and DRG tissues (Supplementary Figure S11A-B).

**Figure 6.**
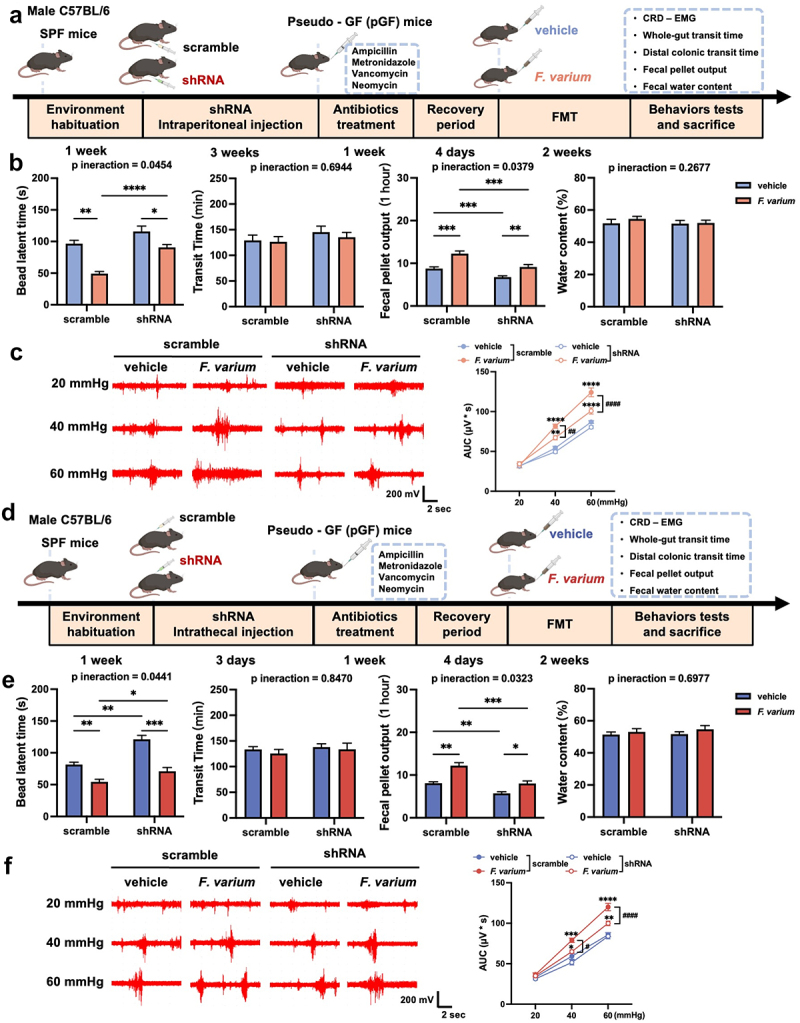
Colon or DRG Piezo2 knockdown partially alleviate visceral hypersensitivity and gut dysmotility induced by *Fusobacterium varium* intervention.

To further investigate this interaction, we performed specific interventions using *F. varium* in Piezo2 KD pGF mice, targeting either the colon (Figure 6(a)) or the DRG (Figure 6(d)). Consistent with our previous findings using FMT, Piezo2 KD in both the colon and DRG had no effect on body weight (Supplementary Figure S12A-B). However, Piezo2 KD partially alleviated the heightened visceral sensitivity, reduced colon transit time, and increased the number of fecal pellets output, without causing significant differences in whole-intestinal transit time or fecal water content (Figures 6(b,c) and (e,f)).

(a). Schematic diagram illustrating the *Fusobacterium varium* intervention in colon Piezo2 KD pGF mice. (b) Effects on colon transit time, whole-intestinal transit time, number of fecal pellet output, and water content of fecal pellet. *n* = 8 mice per group, using two-way ANOVA with Tukey’s post-hoc test. (c) Results of CRD-EMG. *n* = 8 mice per group, using three-way ANOVA with Tukey’s post-hoc test. (d) Schematic diagram depicting the *Fusobacterium varium* intervention in DRG Piezo2 KD pGF mice. (e) Changes in colon transit time, whole-intestinal transit time, number of fecal pellet output, and water content of fecal pellet. *n* = 10 mice per group, using two-way ANOVA with Tukey’s post-hoc test. (f) CRD-EMG results. *n* = 10 mice per group, using three-way ANOVA with Tukey’s post-hoc test. Data are presented as mean ± SEM. *p < 0.05, **p < 0.01, ***p < 0.001, ****p < 0.0001.

### Fusobacterium varium *may regulate Piezo2 via affecting the metabolites of indoles and derivatives*

**Figure 7.**
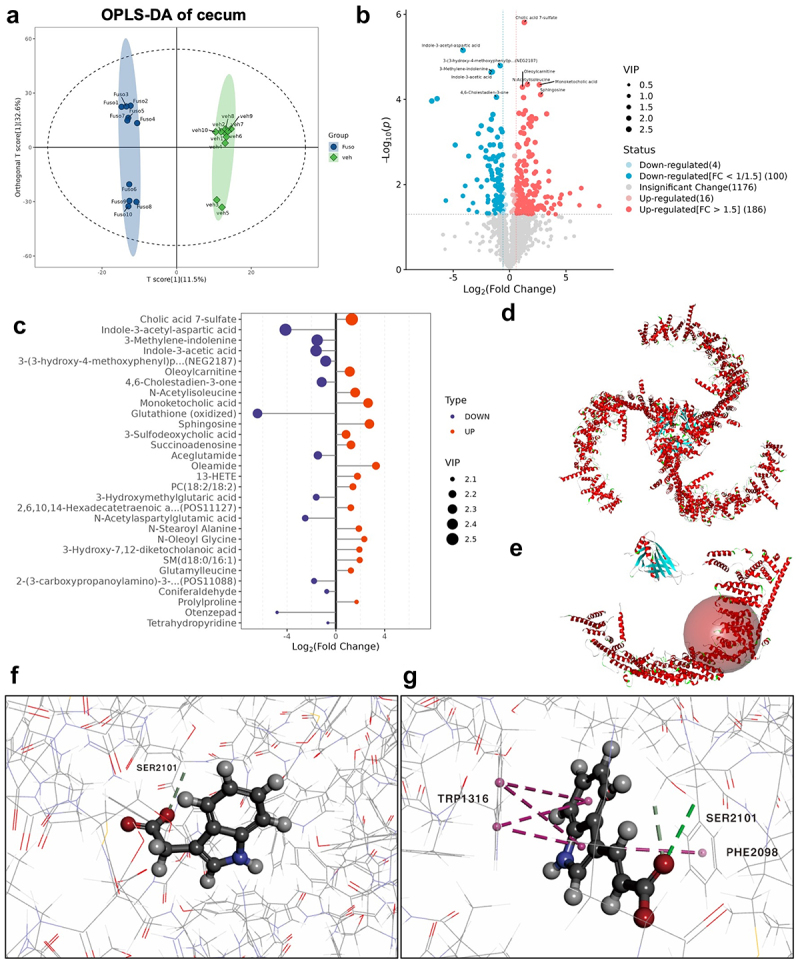
Indole-3-acetic acid and indole-3-acrylic acid emerge as potential key differential metabolites capable of binding to Piezo2.

To gain insights into the underlying mechanism, we conducted an untargeted metabolomics analysis on cecal contents from pGF mice after *F. varium* intervention. Metabolome profiling, using OPLS-DA analysis, revealed distinct metabolic patterns between the control group and the group exposed to *F. varium* (Figure 7, Supplementary Figure S13A). Through volcano plots and bar plots, we identified 290 key metabolites, including Cholic acid 7-sulfate, Indole-3-acetyl-aspartic acid, and 3-Methylene indolenine (Figure 7(a)). A circle heatmap further highlighted the significant differences in the expression of these 290 differential metabolites between the two groups (Supplementary Figure S13B). Importance analysis revealed that indoles and their derivatives, such as Indole-3-acetyl-aspartic acid, 3-Methylene indolenine and Indole-3-acetic acid (IAA), were among the most profoundly affected metabolites (Figure 7(c)).

(a) OPLS-DA analysis results. (b) Volcano plot showcasing cecal differential metabolite. (c) Fold change and VIP score diagrams for the Top 30 differential metabolites. (d) Diagram of the Piezo2 molecular structure model. (e) Illustration of the Piezo2 molecular docking binding domain. (f) Detailed interaction between IAA and Piezo2 residues. (g) Detailed interaction between indole-3-acrylic acid and Piezo2 residues.

To explore potential interactions between Piezo2 and these metabolites, we undertook molecular docking simulations. Employing the molecular structure of Piezo2 retrieved from the Protein Data Bank (PDB) (Figure 7(d)), we predicted binding interactions between Piezo2 and five key indoles and derivatives metabolites (Supplementary Table S4). The alternative binding domain of Piezo2 was referred based on the binding domains of Yoda1 (a Piezo1 agonist) and Piezo1 (Figure 7(e)). The results suggested that Indole-3-acetic acid (IAA) and Indole-3-acrylic acid have the potential to bind to Piezo2 (Figure 7(f-g), Supplementary Figure S14).

## Discussion

The Piezo family, a newly discovered class of mechanosensory proteins, has progressively unveiled its pivotal role in somatic and visceral sensation.^42,43^ Notably, Piezo2 has been implicated in the regulation of GI motility.^15,44^ Previous reports have shown that intestinal microbiota can modulate Piezo1 via microbiota-derived metabolism of ssRNA, subsequently affecting 5-HT release in the intestinal epithelium.^4^ However, the question of whether Piezo2 is regulated by gut microbiota remained unanswered until now. This study marks a pioneering exploration into the regulatory effects of intestinal microbiota on Piezo2. By leveraging both human subjects and animal models, our research sheds light on the complex interplay between intestinal microbiota, Piezo2, and GI functions. Clinical studies initially suggested a correlation between the symptoms of IBS-D patients, gut dysbiosis, and Piezo2 expression. Further FMT and co-housing experiments bolstered the hypothesis that the gut microbiota of IBS-D patients can induce IBS-D-like manifestations, including visceral hypersensitivity and colonic dysmotility, in pGF mice, along with increased Piezo2 expression. By knocking down Piezo2 in the colon and DRG of pGF mice respectively, we observed partial alleviation of the intestinal dysfunction caused by FMT. Subsequent 16S rRNA sequencing and single-bacteria studies identified *F. varium* as the key bacterium. *F. varium* was found to cause intestinal dysfunction by upregulating Piezo2 expression. Metabolomics analysis, coupled with molecular docking, led us to postulate that *F. varium* may regulate Piezo2 through its metabolites, IAA and Indole-3-acrylic acid, which have the potential to bind to Piezo2, thereby impacting visceral sensitivity and colonic motility.

The findings from both clinical studies and animal experiments underscore the associations between Piezo2 protein levels in the colon and DRG with alterations in intestinal microbiota, and their role in visceral hypersensitivity and gut dysmotility induced by intestinal dysbiosis. Our clinical research indicates that IBS-D patients exhibit visceral hypersensitivity and intestinal dysmotility, accompanied by intestinal dysbiosis. Immunofluorescence analysis revealed a lower ratio of Piezo2^+^ cells to 5-HT^+^ cells in the sigmoid colon mucosa of IBS patients compared to HC. Interestingly, this ratio negatively correlated with the pressure value (mmHg) obtained during the barostat test, indicating a positive correlation with visceral sensitivity. However, due to limitations in tissue availability, we could not assess Piezo2 protein level in the entire layer of colon. As a result, immunofluorescence staining provides a partial view of Piezo2 level in the colonic mucosa.

Although the technology of FMT still requires refinement, it remains a widely employed method for investigating gut microbiota. Numerous previous studies^45–48^ have confirmed that FMT administered via oral gavage can successfully transfer patients’ gut microbiota into the intestines of experimental animals. In the animal model of intestinal dysfunction induced by intestinal dysbiosis that we established through FMT, there was a notable increase in Piezo2 protein levels in both the colon and DRG, accompanied by a significant elevation in serum 5-HT levels. These changes are believed to contribute, either directly or indirectly, to intestinal dysfunction. Recent studies have shown that individuals with Piezo2 deficiency experience a variety of GI disturbances (such as lumps and watery stools)^15^ compared to the general population. Another study found that mechanical stimulation-induced 5-HT release and Piezo2 expression in ECs cells declined with age.^44^ Furthermore, mouse-specific knockout of Piezo2 in *Tph1*^+^ EEC cells resulted in a significant prolongation of colonic and overall digestive tract transit time.^44^ 5-HT, as a crucial neurotransmitter, has been extensively studied in relation to IBS.^49,50^ Simultaneously, Piezo2 plays a pivotal role in mediating the release of 5-HT from enterochromaffin cells.^8,9^ We further established Piezo2 as a crucial molecule in visceral hypersensitivity and colonic dysmotility induced by intestinal dysbiosis by knockdown Piezo2 in the colon or DRG. Previous studies have found that knocking down Piezo2 in rat DRG neurons significantly alleviates visceral hypersensitivity caused by acetic acid enema.^10^ Comparable observations were made in the 2,4,6-trinitrobenzene sulfonic acid (TNBS)-induced post-inflammatory (PI)-IBS mouse model.^21^ However, our study distinguished itself by employing an IBS model induced by FMT, which provides a unique perspective compared to chemical-induced (acetic acid/TNBS) IBS model. This FMT induced IBS model allows for a direct exploration of the interaction between intestinal microbiota and Piezo2.

After establishing Piezo2 as a crucial molecule in intestinal dysfunction stemming from dysbiosis, we conducted fecal 16S rRNA sequencing analysis to identify potential key bacterial genera involved. Through this analysis, we observed the impact of specific bacteria on intestinal function and Piezo2 expression. By administering *A. muciniphila* or *F. varium* to pGF mice, we gained deeper insights into their respective effects on GI function. Our findings indicate that *A. muciniphila* may have a beneficial effect on gut dysmotility and visceral hypersensitivity, whereas *F. varium* appears to exacerbate these manifestations, consistent with the results observed in FMT-IBS mice. Our findings indicated that at the phylum level, the proportion of p_Fusobacteria in IBS-D patients (1.936%) was 4.5 times higher than that in HC (0.427%). At the genus level, the proportion of *g_Fusobacterium* in IBS-D patients (1.827%) was nearly 71.5 times higher than that in HC (0.026%). This observation aligns with several studies that have reported the prominence of *g_Fusobacterium* in IBS-D patients.^40,41,51–53^ Furthermore, elevated *g_Fusobacterium* levels were also observed in the IBS-D donors (Supplementary Figure S4G) and the mice that received FMT from IBS-D patients (Supplementary Figure S9I). Therefore, we hypothesize that *g_Fusobacterium* may be an important genus contributing to visceral hypersensitivity and colonic dysmotility.

Furthermore, our study has demonstrated that Piezo2 may constitute a significant pathway contributing to the colonic dysmotility and visceral hypersensitivity induced by *F. varium*. This conclusion was further reinforced by experiments wherein Piezo2 was knocked down in both the colon and DRG, revealing that *F. varium* partially exerts its effects through this molecule. *F. varium*, a bacterial species implicated in various human diseases and conditions, has been observed in higher abundance in colorectal cancer tissue relative to adjacent normal tissue, hinting at a potential role in cancer development.^54^ Our study also uncovered a positive correlation between *F. varium* and the levels of IL-1β and IL-6 in patients with cerebral autosomal-dominant arteriopathy with subcortical infarcts and leukoencephalopathy (CADASIL). Additionally, infection with *F. varium* isolated from these patients triggered systemic inflammation and behavioral disorders in *Notch3*^R170C/+^ mice.^55^ Genomic analysis of *F. varium* Fv113-g1 revealed multiple virulence factors, specifically including paralogs of the type V secretion system (T5SS) and fusobacterial adhesion (FadA), which contribute to underlying mucosal inflammation.^56^

Based on these findings, we conducted an untargeted metabolomics analysis to elucidate the potential mechanism by which *F. varium* interacts with Piezo2. This analysis was complemented by molecular docking to predict which differential metabolites could bind to Piezo2. Previous studies have indicated that IAA, produced by intestinal microbe-driven tryptophan metabolism, acts as a protein-bound uremic toxin. Hemodialysis patients exhibit elevated levels of IAA, and *F. varium* is enriched in individuals with high serum IAA levels.^57^ Among the metabolites identified through our untargeted metabolomics analysis, several indoles and their derivatives emerged as significant. Molecular docking predictions revealed that both indole-3-acrylic acid and IAA can bind to Piezo2, hinting that these compounds might play crucial roles in interacting with Piezo2 and potentially mediating *F. varium* ability to elevate Piezo2 protein levels.

However, it is important to acknowledge that the observed changes in IAA and indole-3-acrylic acid levels still necessitate further validation through metabolomics analysis on patients with IBS-D and HCs following an expansion of the sample size. Meanwhile, we recognize that there are some innovative methods, such as the measurement of whole intestinal transit time,^58,59^ that could be employed in future studies. Also, FMT, as a commonly used method in microbiome research, also requires refinement in future investigations.

In summary, this study pioneers the establishment that Piezo2 is modulated by the gut microbiota, which in turn exerts its effects on visceral sensitivity and colonic motility. Notably, *F. varium* potentially regulates Piezo2 expression by modulating indole metabolite levels, thereby impacting visceral sensitivity and colonic motility. These findings underscore the intricate relationship between gut microbiota, Piezo2, and GI function in the development of IBS-D. Moreover, exploring therapeutic strategies that manipulate gut microbiota composition to regulate Piezo2 appears promising for managing visceral hypersensitivity and gut dysmotility.

## Supplementary Material

Supplemental Material

## Data Availability

All amplicon sequencing data have been deposited in the National Genomics Data Center (NGDC)^60,61^ and are accessible under BioProject accession number PRJCA035313 for human sample (https://bigd.big.ac.cn/gsa-human/browse/HRA010166.) and PRJCA035312 for mouse sample (https://bigd.big.ac.cn/gsa/browse/CRA022432). Additionally, the untargeted metabolism data have been deposited in the NGDC and can be found under BioProject accession number PRJCA035357 (https://ngdc.cncb.ac.cn/omix/release/OMIX008747).
