# Supplementary material for: Mechanosensory Piezo2 regulated by gut microbiota participates in the development of visceral hypersensitivity and intestinal dysmotility
Source: Gut Microbes. 2025 Apr 28;17(1):2497399. doi: 10.1080/19490976.2025.2497399 (PMC12045567; doi:10.1080/19490976.2025.2497399)
Supplement: Supplemental Material [file KGMI_A_2497399_SM6274.docx]

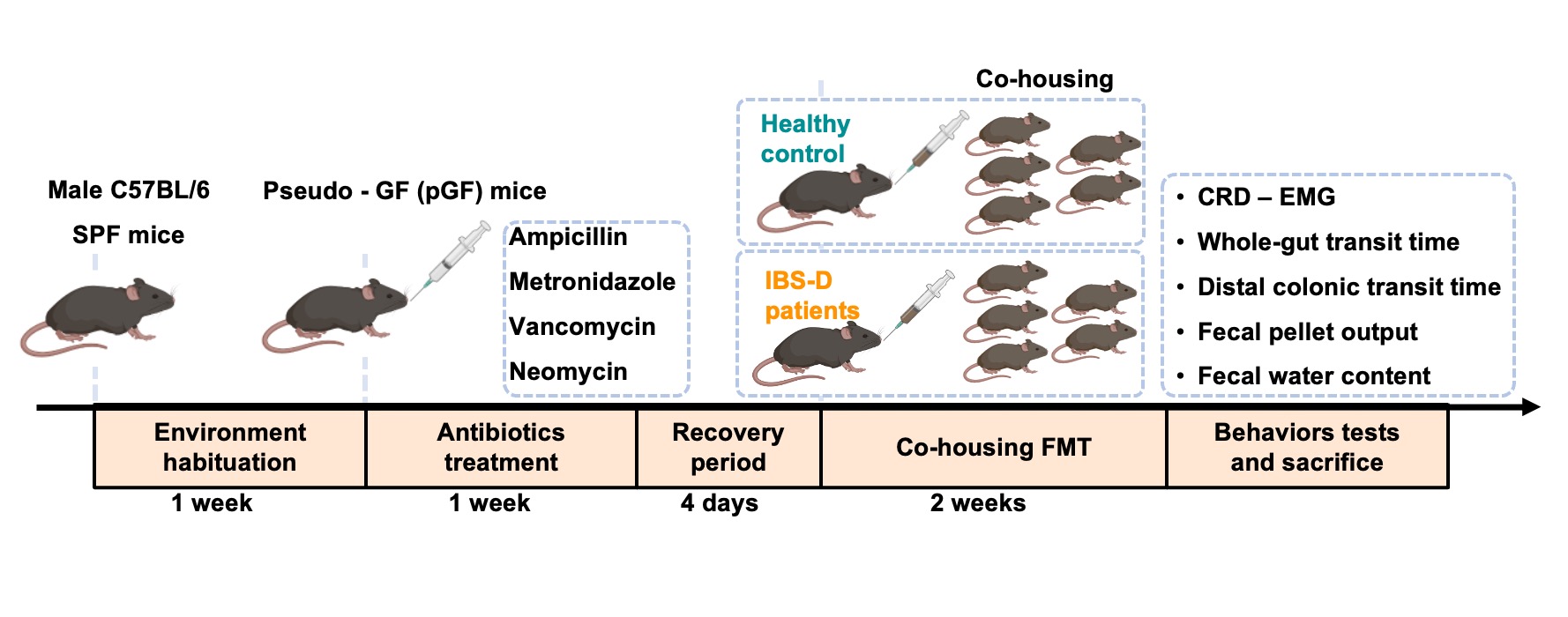


**Supplementary Figure 1. Schematic diagram of IBS model established through Co-housing with FMT in pGF mice.**


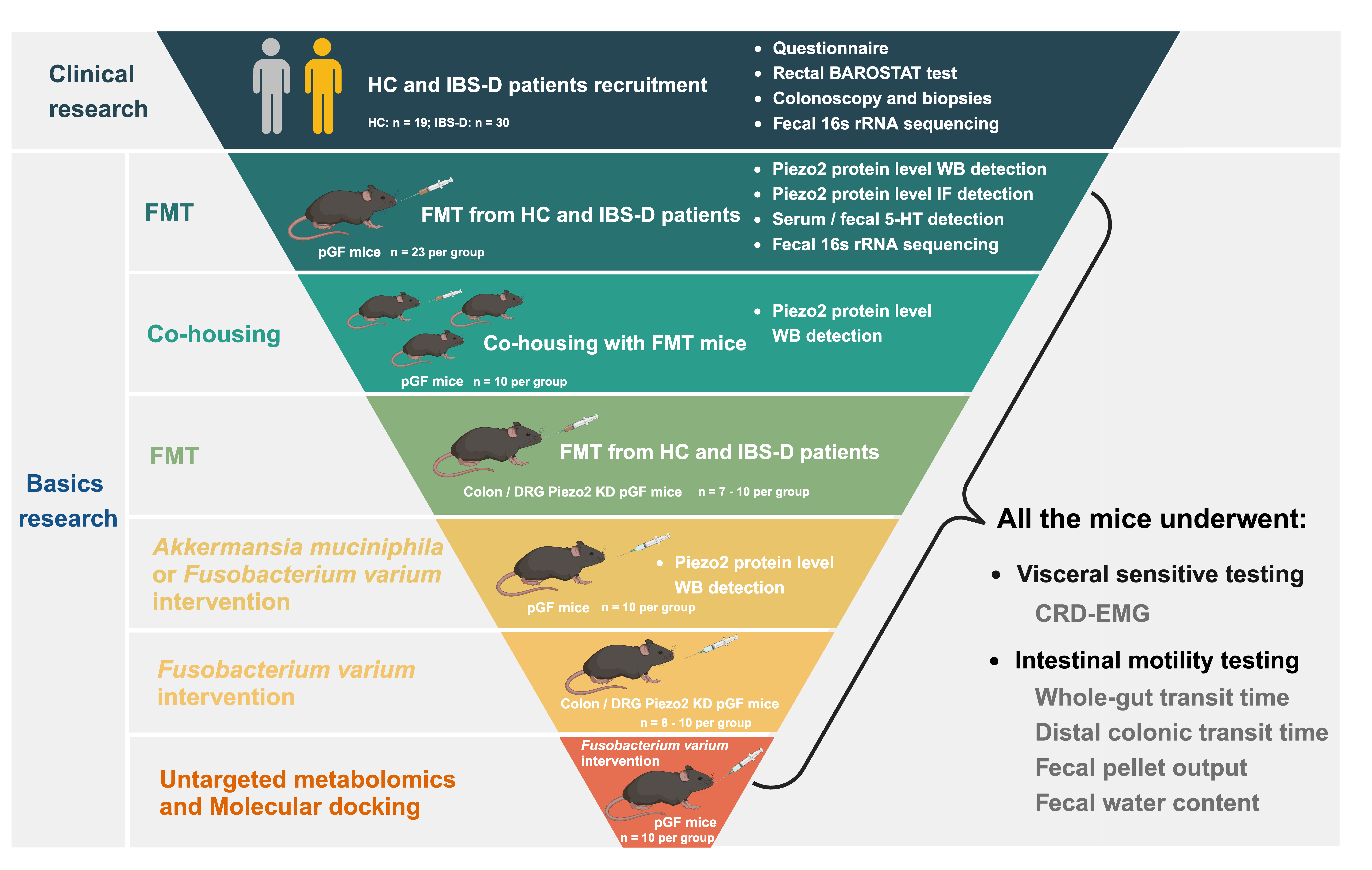


**Supplementary Figure 2. The flowchart of the study.**


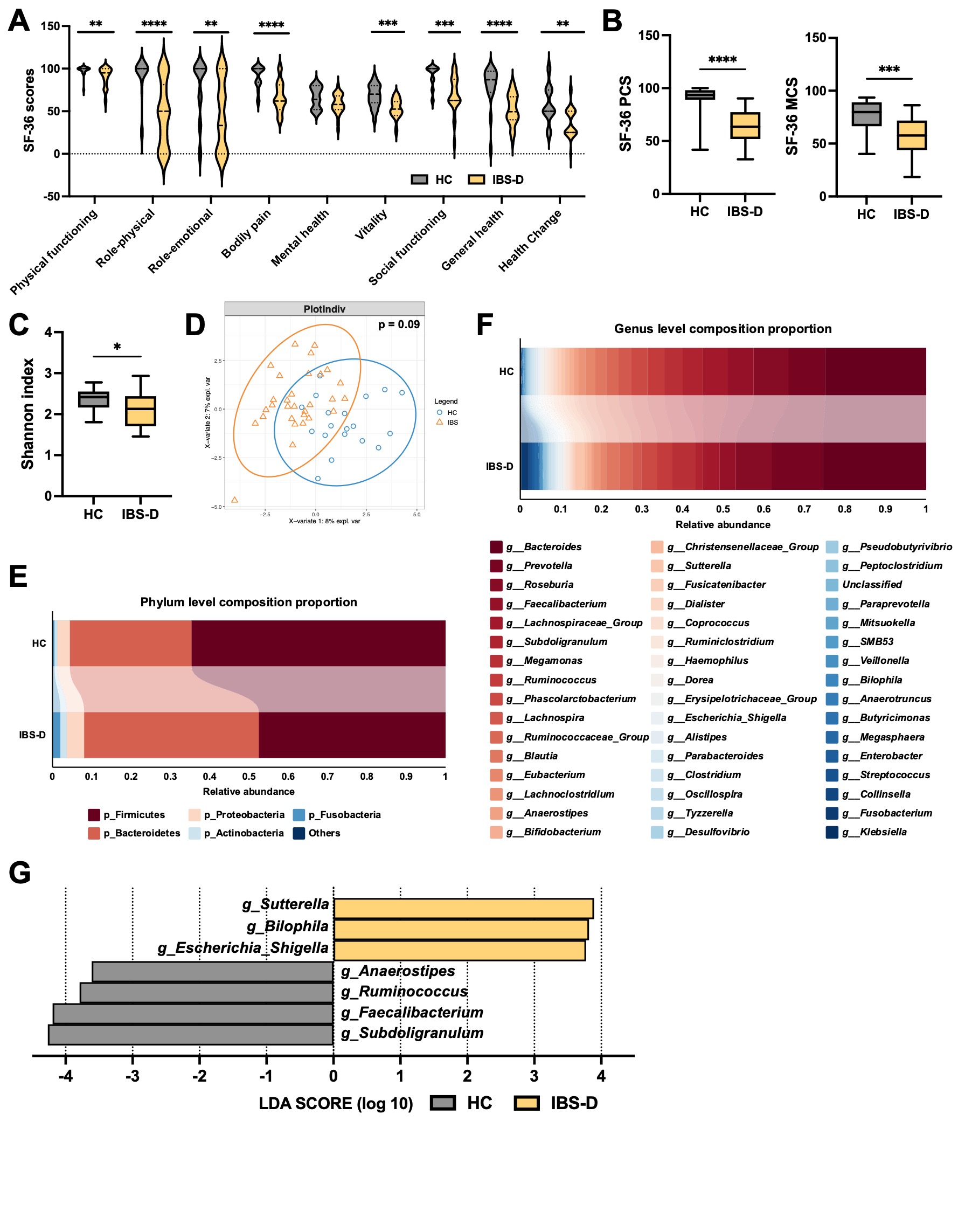


**Supplementary Figure 3.** **Reduced quality of life and intestinal dysbiosis in IBS-D patients.**

A-B) Results of the SF-36 questionnaire. C) α-diversity at genus level. D) PLS-DA score plot at the genus level. E) Composition of the two groups at the phylum level. F) Composition of the two groups at the genus level. G) Differential abundance of gut microbiota. Data are presented as median ± quartiles. HC, n = 19; IBS-D, n = 30. Nonparametric test with Mann-Whitney test. **p < 0.01, ***p < 0.001, ****p < 0.0001.

(Supplementary Figure 4F).


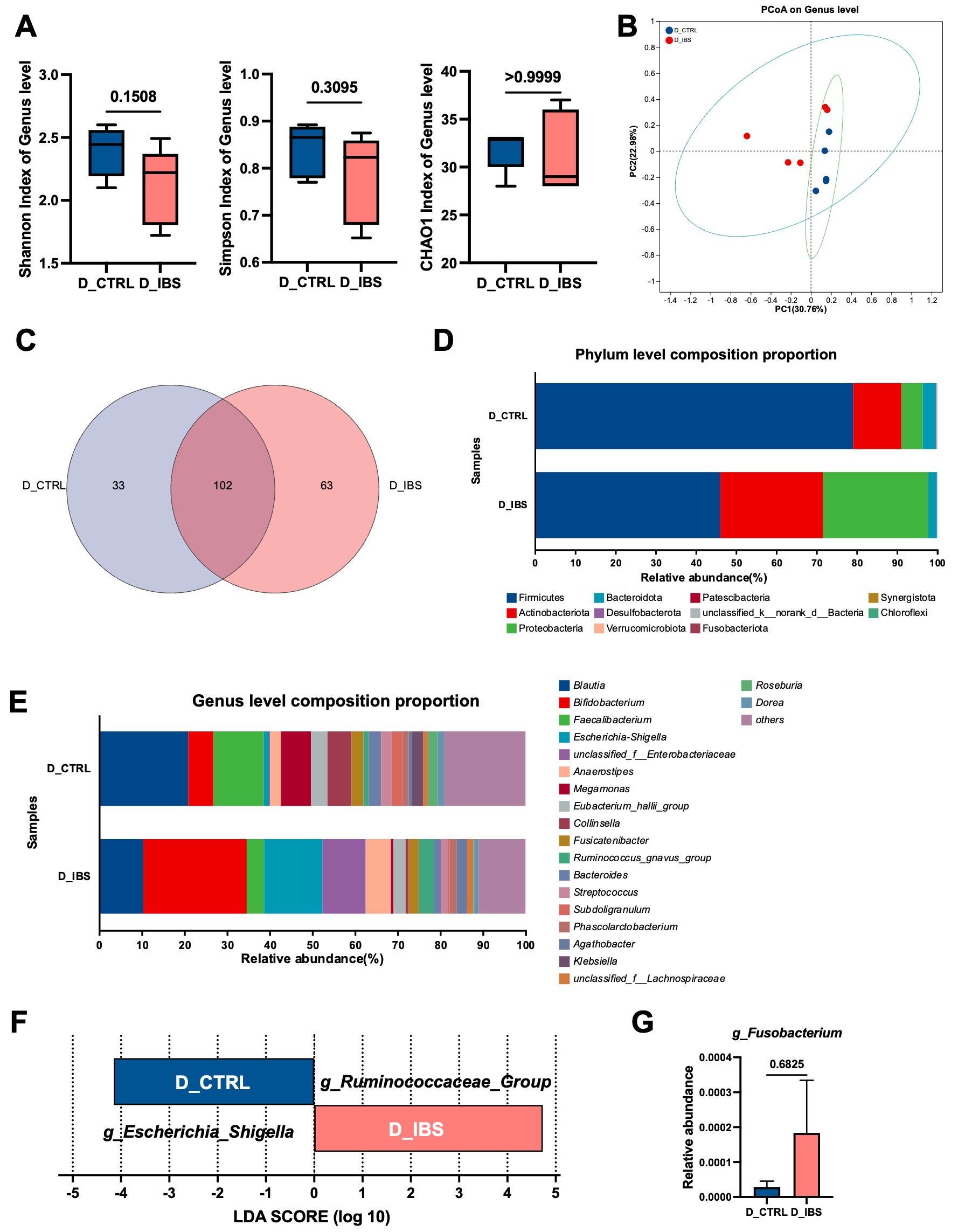


**Supplementary Figure 4.** **Gut microbiota analysis of FMT donors.**

A) α-diversity index. B) PCoA analysis. C) Venn diagram representation. D) Compositional analysis at the phylum level for two groups. E) Compositional analysis at the genus level for two groups. F) Differential abundance of gut microbiota. G) Relative abundance of *g_Fusobacterium*. Data are presented as median ± quartiles. HC, n = 5; IBS-D, n = 5. Statistical analysis was performed using the nonparametric test with the Mann-Whitney test.

**
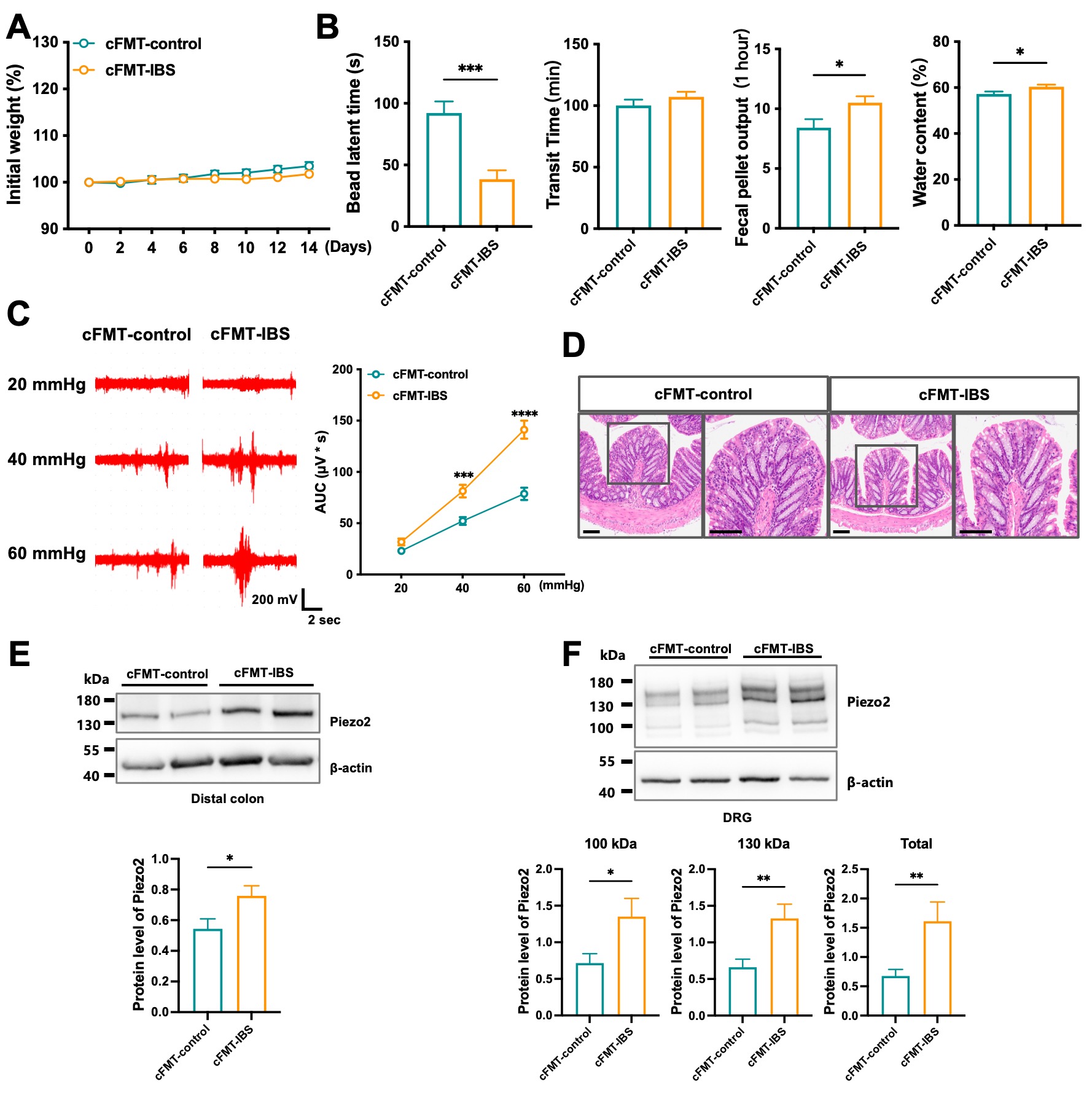
**

**Supplementary Figure 5. Co-housing with FMT-IBS pGF mice induces visceral hypersensitivity, gut dysmotility and upregulates of Piezo2 in pGF mice.**

A) Body weight of the mice. B) Colon transit time, whole-intestinal transit time, number of fecal pellet output, and water content of fecal pellet. C) Results of the CRD-EMG. D) H&E staining of the distal colon of pGF mice after co-housed with FMT-treated mice. E) Piezo2 protein level in the distal colon. F) Piezo2 protein level in DRG neurons. Data are presented as mean ± SEM. n = 10 mice per group, using unpaired t-test or two-way ANOVA with Sidak’s post-hoc test. Scale bar = 100 μm. *p < 0.05, **p < 0.01, ***p < 0.001, ****p < 0.0001.


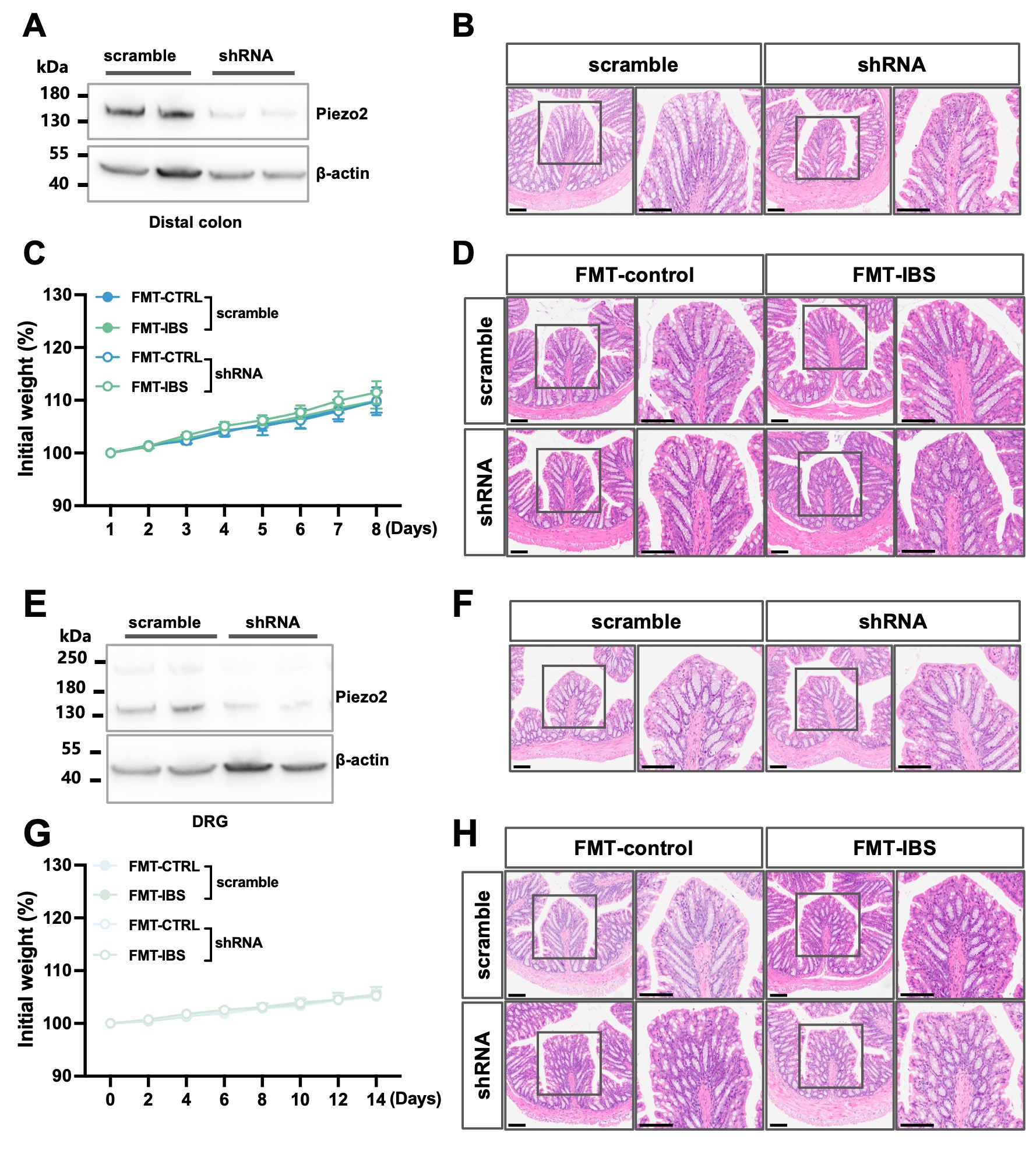


**Supplementary Figure 6. Body weight changes and colon H&E staining in colon or DRG Piezo2 KD mice.**

A) Protein level of Piezo2 in the distal colon following intraperitoneal injection of Piezo2-shRNA in SPF mice. B) H&E staining of the colon after intraperitoneal injection of Piezo2-shRNA in SPF mice. C) Body weight changes during FMT from pGF mice. n = 8 mice per group; using three-way ANOVA with Tukey’s post-hoc test. D) H&E staining of the colon after FMT in colon Piezo2 KD pGF mice. E) Protein level of Piezo2 in DRGs after intrathecal injection of Piezo2-shRNA in SPF mice. F) H&E staining of the colon after intrathecal injection of Piezo2-shRNA in SPF mice. G) Body weight changes during FMT from pGF mice. n = 7-10 mice per group; using three-way ANOVA with Tukey’s post-hoc test. H) H&E staining of the colon after FMT in DRG Piezo2 KD pGF mice. Data are presented as mean ± SEM. Scale bar = 100 μm.


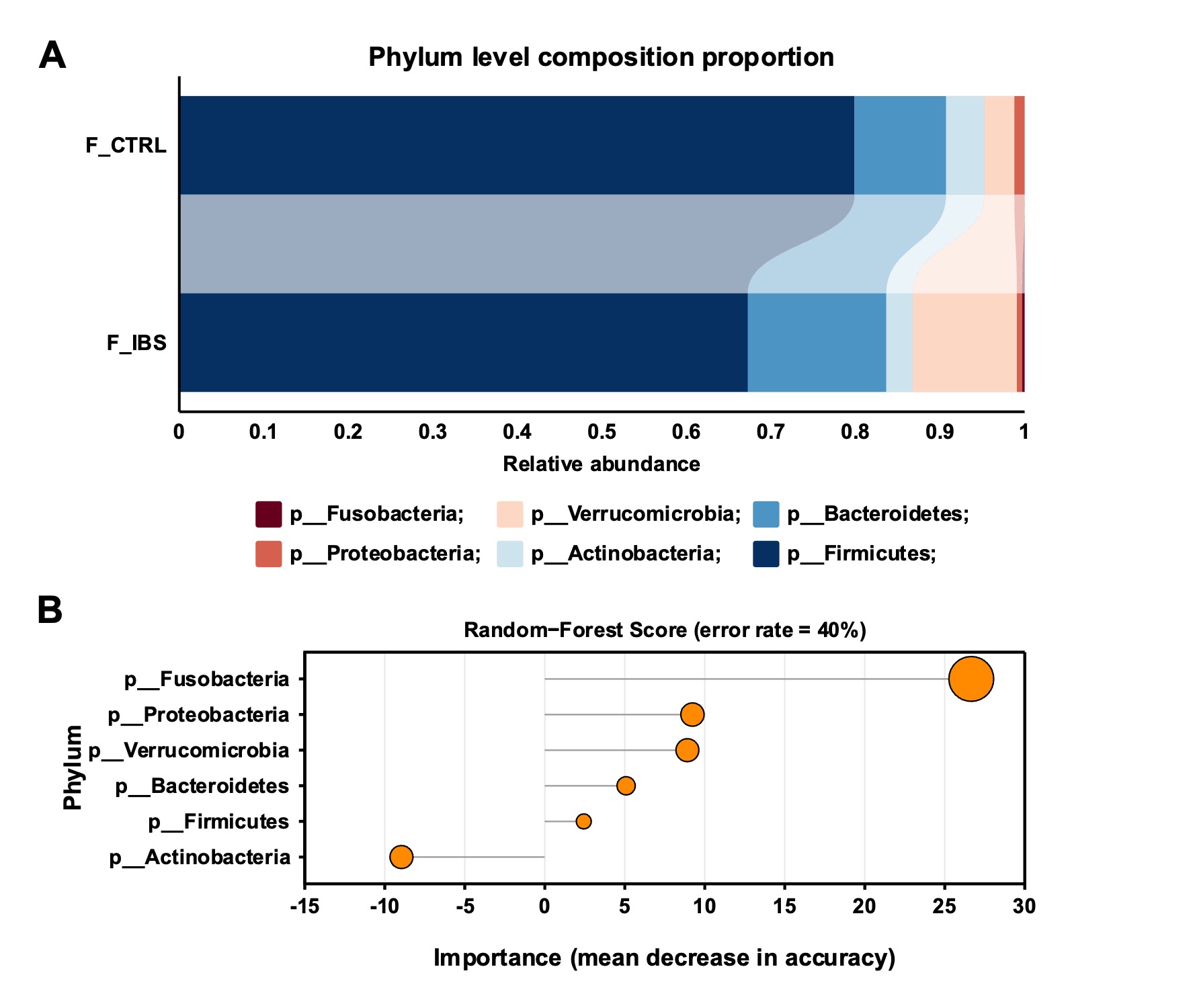


**Supplementary Figure 7.** **Phylum level composition of 16S rRNA sequencing in pGF mice after FMT.**

A) Phylum-level composition of the two groups of mice. B) Supervised random forest analysis at the phylum level.


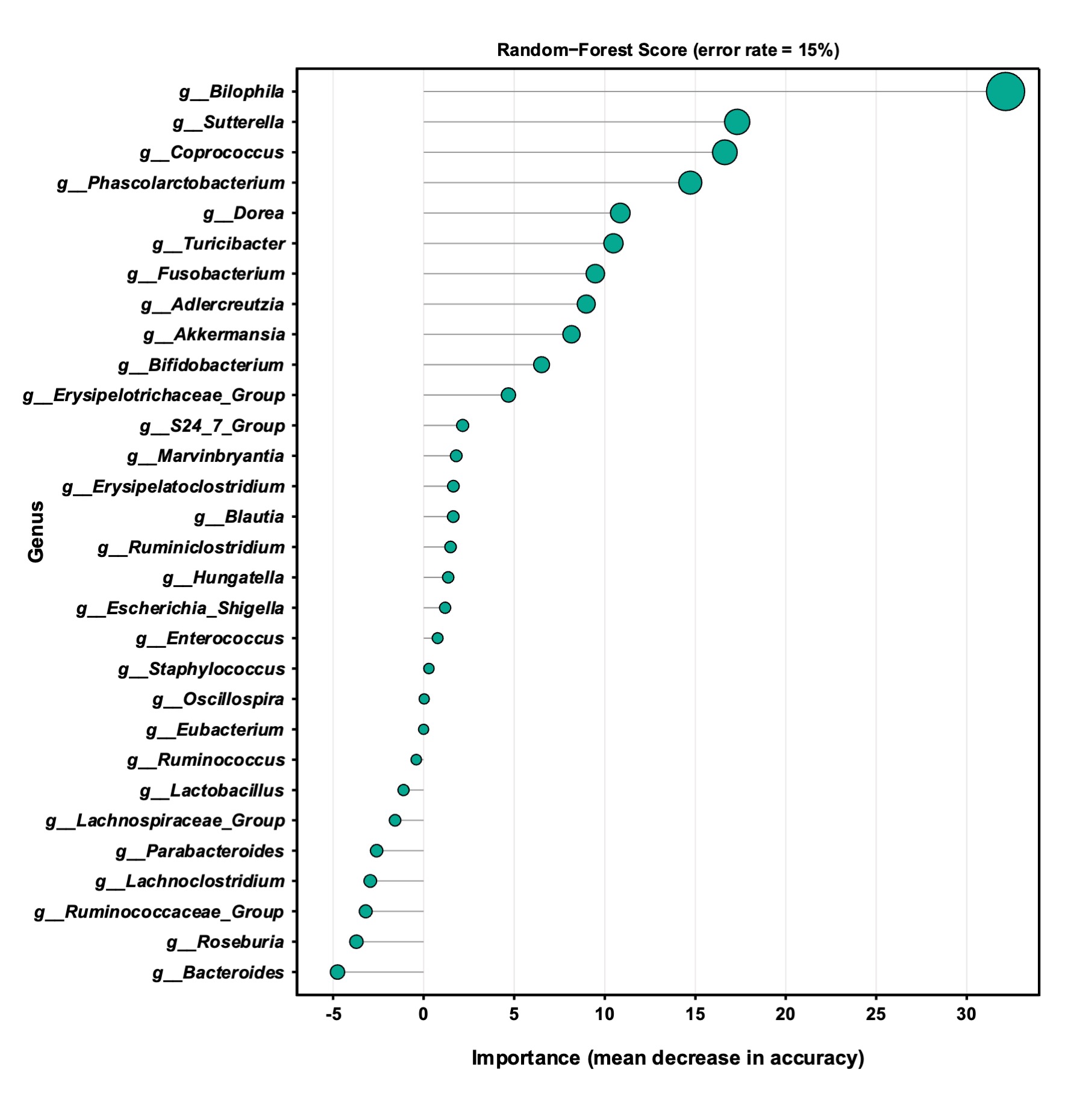


**Supplementary Figure 8. Supervised random forest analysis at the genus level.**

The supervised random forest analysis identified *g_Bilophila*, *g_Sutterella* and *g_Coprococcus* as the top three contributors to the classification of microbiota status.


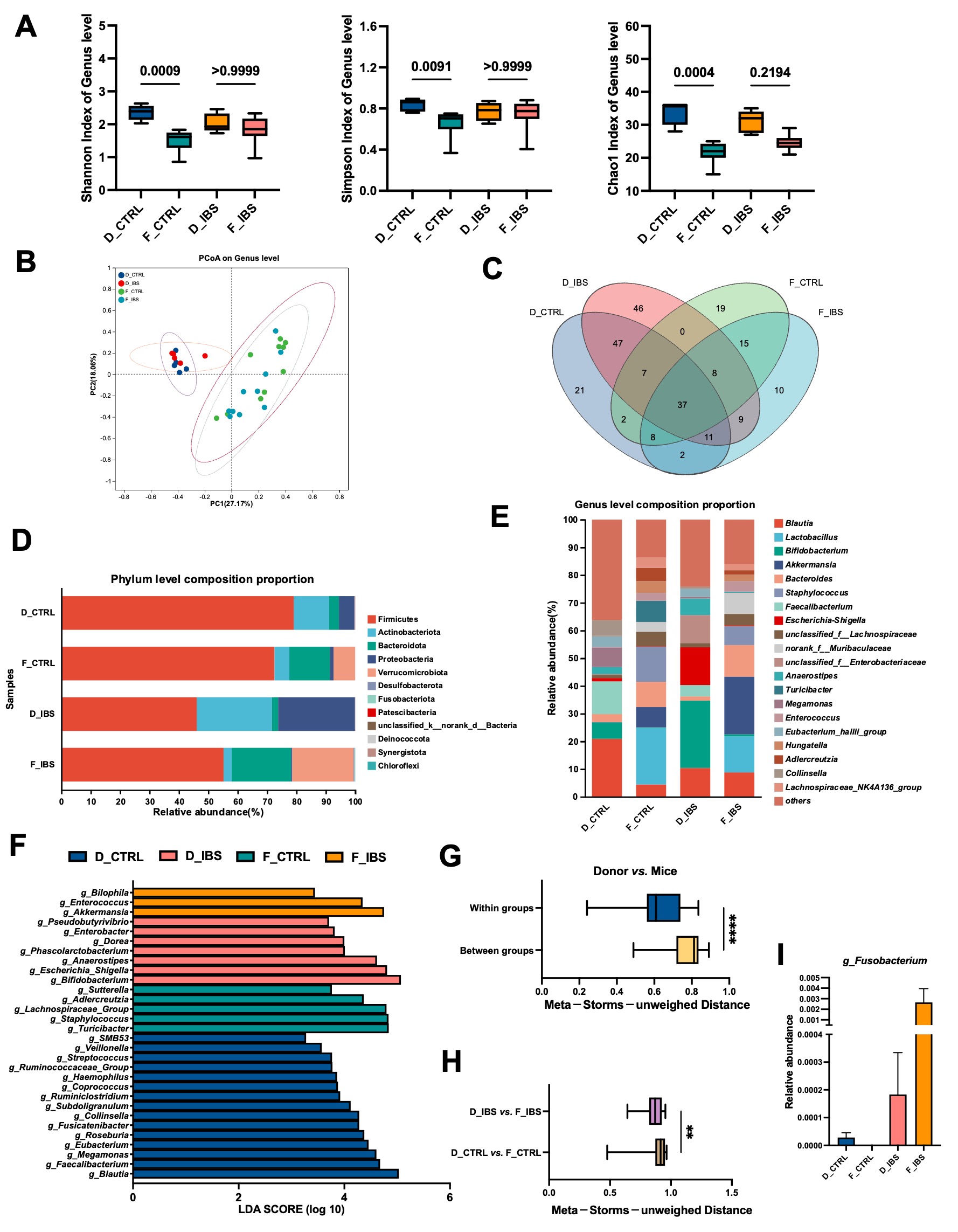


**Supplementary Figure 9. Comparative analysis of gut microbiota between donors and FMT-recipient mice.**

A) α-diversity. B) PCoA analysis. C) Venn diagram representing unique and shared OTUs. D) Phylum-level composition of the four groups. E) Genus-level composition of the four groups. F) Differential abundance of gut microbiota. G) Distance analysis between donors and mice. H) Distance analysis among the four groups. I) Relative abundance of *g_Fusobacterium*. Data are presented as median ± quartiles. Sample sizes: D_CTRL, n = 5; D_IBS, n = 5; F_CTRL, n = 10; F_IBS, n = 10. Using nonparametric test with Wilcoxon test or Mann-Whitney test. **p < 0.01, ****p < 0.0001.


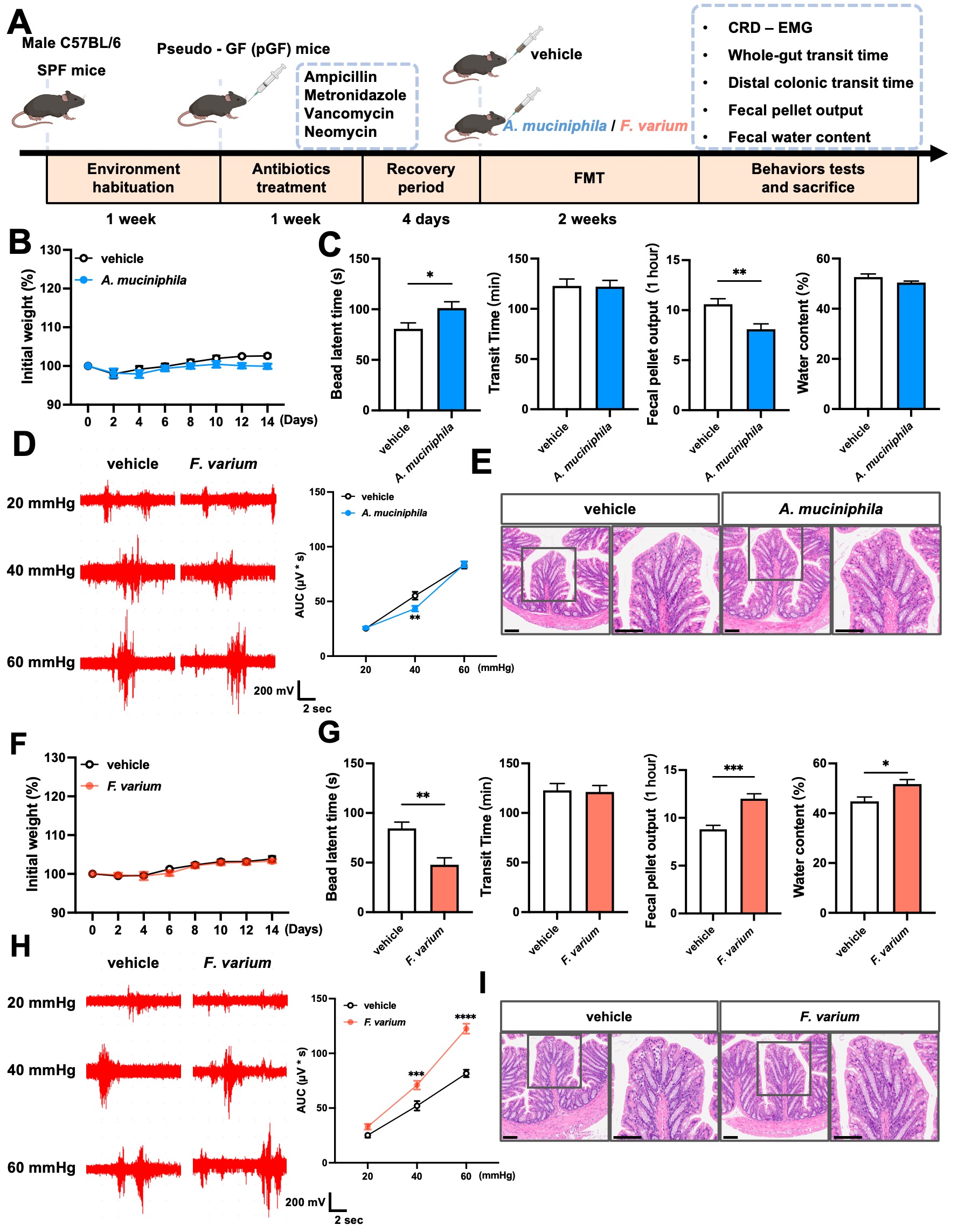


**Supplementary Figure 10. Oral gavage with *Fusobacterium varium*, rather than *Akkermansia muciniphila*, induced visceral hypersensitivity and gut dysmotility in pGF mice.**

A). Schematic diagram illustrating the intervention with *F. varium* or *A. muciniphila* in pGF mice. B) Body weight changes during the *A. muciniphila* intervention period. C) Colon transit time, whole-intestinal transit time, number of fecal pellet outputs, and water content of fecal pellets after *A. muciniphila* intervention. D) CRD-EMG results following *A. muciniphila* intervention. E) H&E staining of the diatal colon after *A. muciniphila* intervention. F) Body weight changes during the *F. varium* intervention. G) Colon transit time, whole-intestinal transit time, number of fecal pellet outputs, and water content of fecal pellet after *F. varium* intervention. H) CRD-EMG results following *F. varium* intervention. I) H&E staining of the distal colon after *F. varium* intervention. Data are presented as mean ± SEM. n = 10 mice per group, using two-way ANOVA with Sidak’s post-hoc test or nonparametric test with Mann-Whitney test. Scale bar = 100 μm. *p < 0.05, **p < 0.01, ***p < 0.001, ****p < 0.0001.


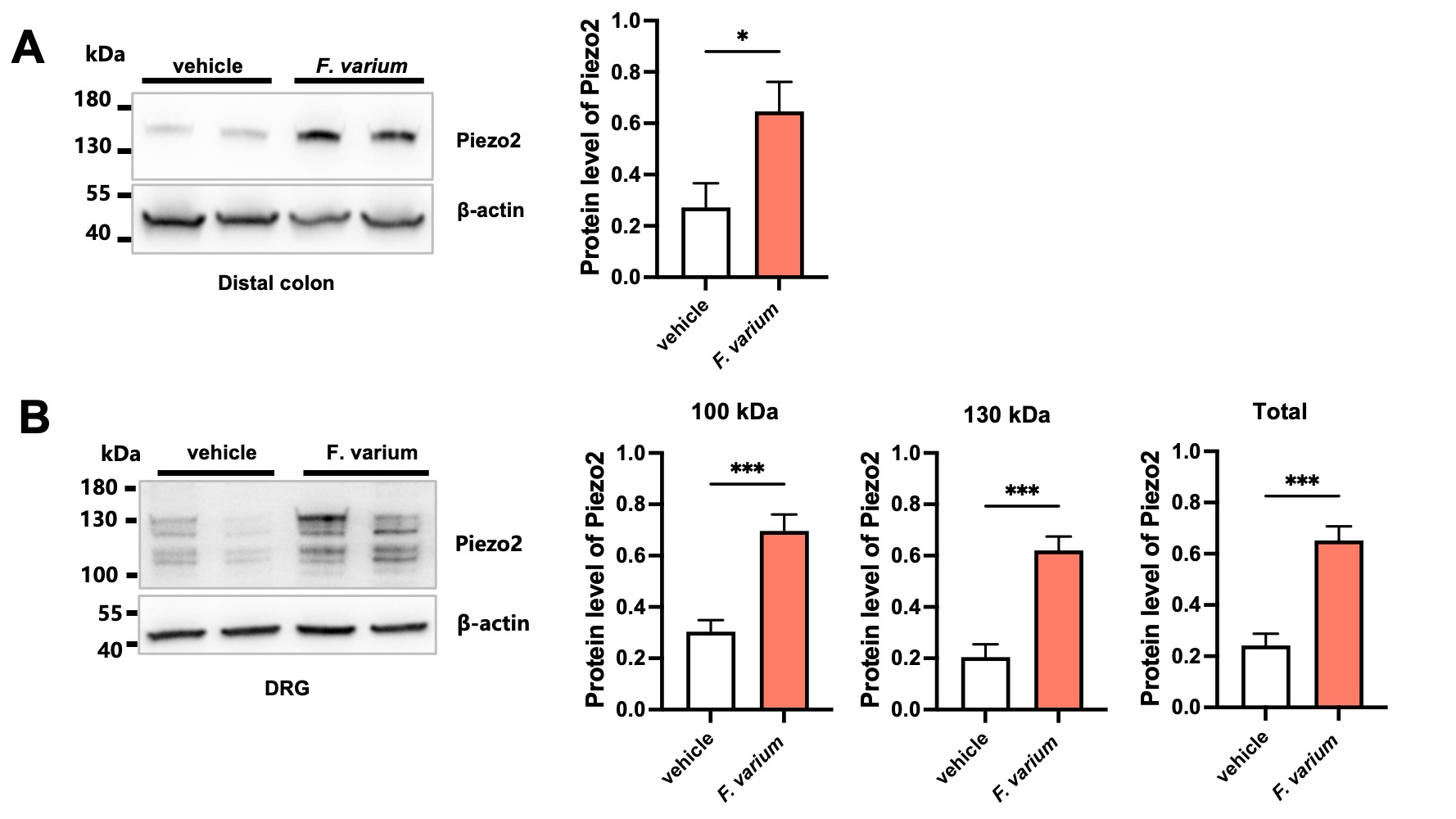


**Supplementary Figure 11. Upregulation of colon and DRG Piezo2 in the pGF mice following *Fusobacterium varium* intervention.**

A) Protein levels of Piezo2 in the distal colon after *F. varium* intervention. B) Protein levels of Piezo2 in DRG neurons after *F. varium* intervention. Data are presented as mean ± SEM. n = 8 mice per group, using nonparametric test with Mann-Whitney test. *p < 0.05, ***p < 0.001.


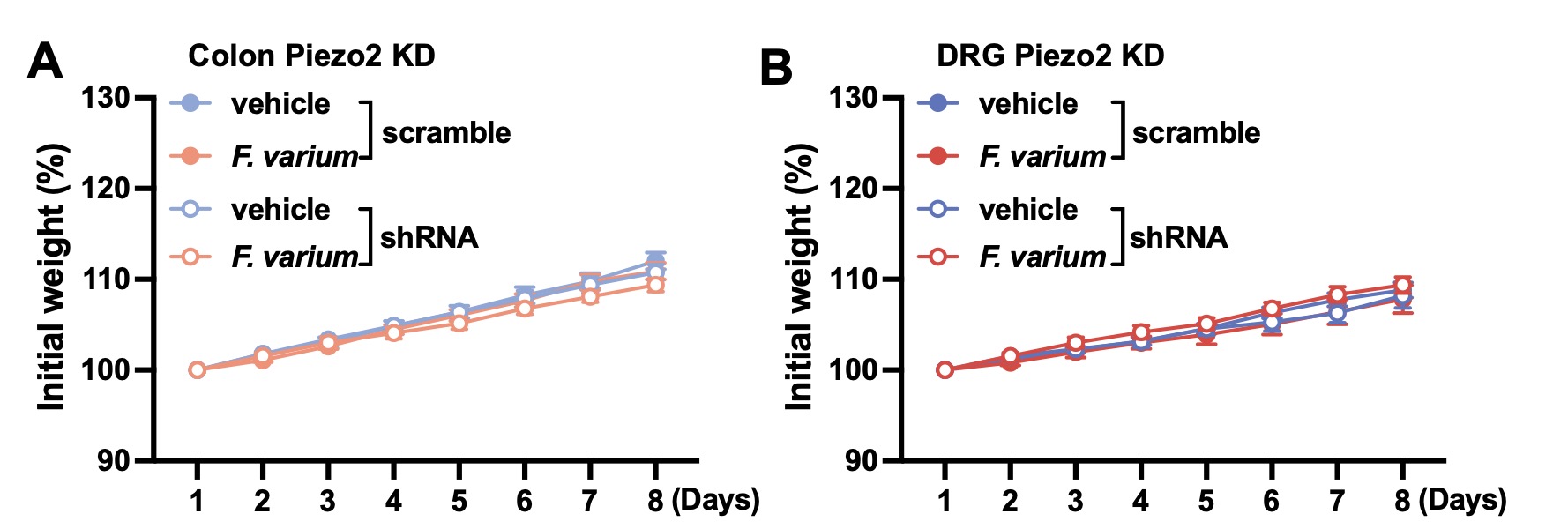


**Supplementary Figure 12. *Fusobacterium varium* intervention did not alter weight change in colon or DRG Piezo2 knockdown pGF mice.**

A) Body weight of colon Piezo2 KD pGF mice following *F. varium* intervention. n = 8 mice per group. B) Body weight of DRG Piezo2 KD pGF mice after *F. varium* intervention. n = 10 mice per group. Data are presented as mean ± SEM, analyzed using three-way ANOVA with Tukey’s post-hoc test.


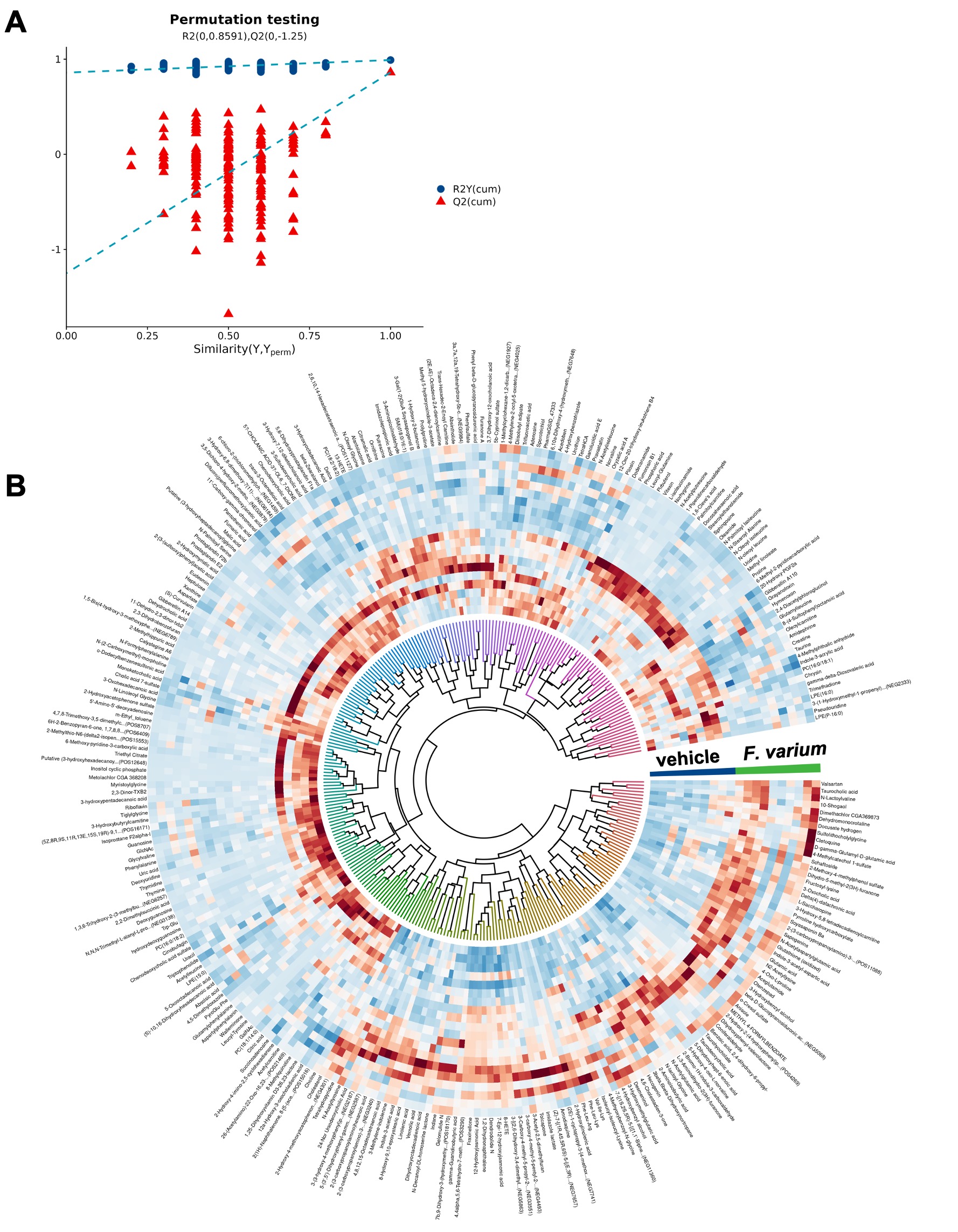


**Supplementary Figure 13. Significant changes in the metabolome of pGF mice after intervention with *Fusobacterium varium*.**

A) Permutation test results for OPLS-DA model validation. B) Circular heatmap analysis.

**
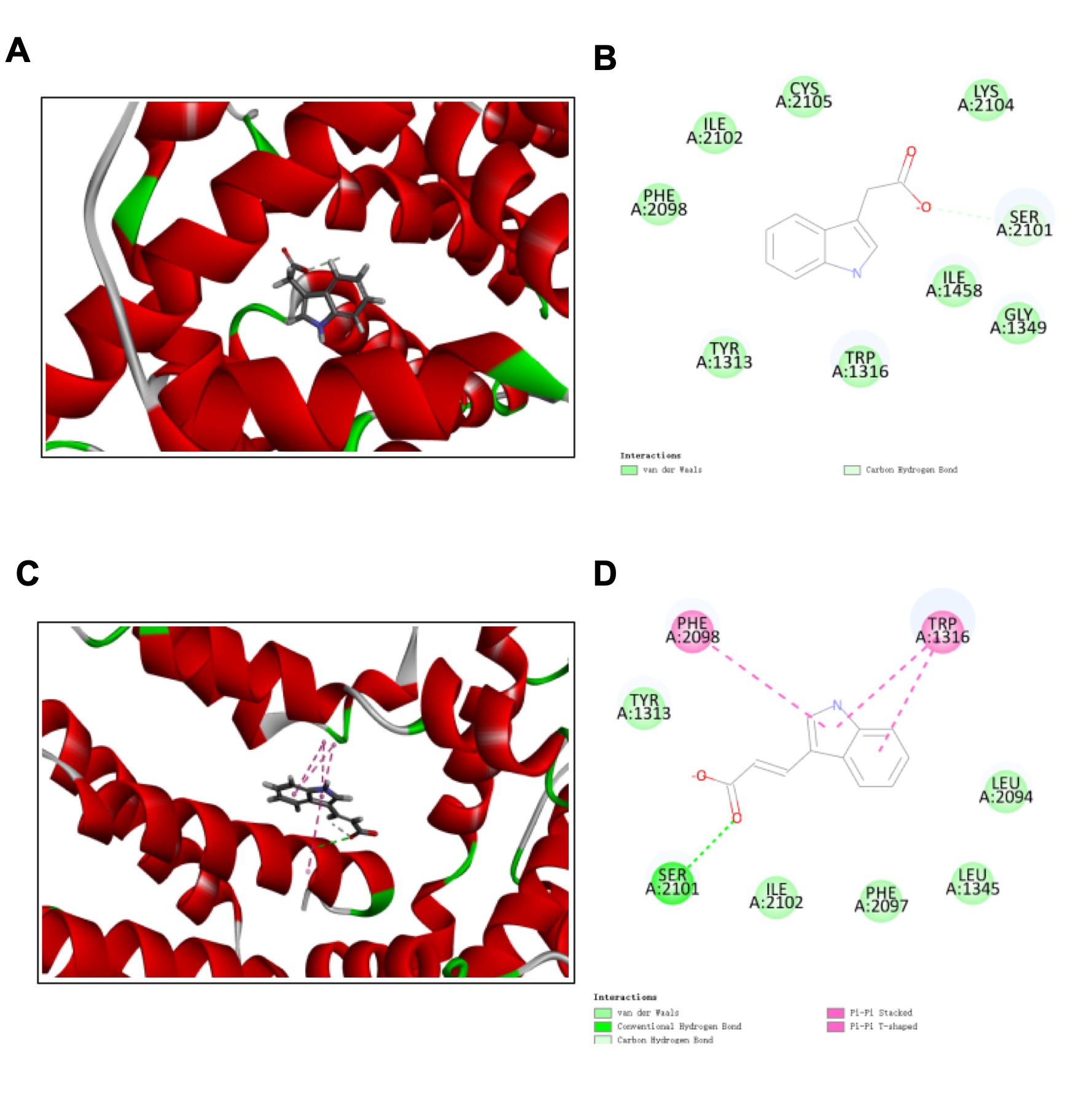
**

**Supplementary Figure 14: Molecular docking suggests that indole-3-acetic acid and indole-3-acrylic acid have the potential to bind to Piezo2.**

A) Binding properties of IAA with the Piezo2 protein. B) 2D diagram of the interaction between IAA and Piezo2. C) Binding properties of indole-3-acrylic acid with the Piezo2 protein. D) 2D diagram of the interaction between indole-3-acrylic acid and Piezo2.

**Supplementary Table S1: Basic characteristics of the subjects among the healthy controls and IBS-D groups**

| **Characteristics** | **Health controls**  (n = 19) | **IBS-D**  (n = 30) | ***P* value** |
| --- | --- | --- | --- |
| Age (year) | 29.21 ± 1.86 | 32.13 ± 1.62 | 0.237 |
| Gender [n (%)] |  |  | 0.770 |
| Male | 17 (89.5%) | 26 (86.7%) |  |
| Female | 2 (10.5%) | 4 (13.3%) |  |
| Height (m) | 1.73 ± 0.01 | 1.74 ± 0.01 | 0.565 |
| Weight (kg) | 68.74 ± 2.92 | 65.48 ± 2.03 | 0.479 |
| BMI (kg/m^2^) | 22.91 ± 0.77 | 21.65 ± 0.55 | 0.226 |
| Smoking [n (%)] | 1 (5.3%) | 5 (16.7%) | 0.235 |

Data are shown as the Mean ± SEM or the number (percentage) for categorical data. Two-tailed non-parametric test (Mann-Whitney) or Chi-square test. IBS-D, Diarrhea-predominant irritable bowel syndrome; BMI, Body mass index.

**Supplementary Table S2: The silence sequence targeting the Piezo2 genes**

|  | **Silence sequence** |
| --- | --- |
| Piezo2-sh1 | GCACCTGATTGGACTTTATTTCAAG  AGAATAAAGTCCAATCAGGTGCTTTTTT |
| Piezo2-sh2 | GCAGAACCATTGTTAAGAATTCAA  GAGATTCTTAACAATGGTTCTGCTTTTTT |
| Piezo2-sh3 | GGACTTAGATGGAGAAGAATTCAA  GAGATTCTTCTCCATCTAAGTCCTTTTTT |
| Piezo2-sh4 | GCTGTGTACTTCTTTGTATTTCAAG  AGAATACAAAGAAGTACACAGCTTTTTT |

**Supplementary Table S3: Basic characteristics of the subjects among the FMT donors**

| **Characteristics** | **Health controls**  (n = 5) | **IBS-D**  (n = 5) | ***P* value** |
| --- | --- | --- | --- |
| Age (year) | 37.20 ± 0.80 | 31.40 ± 3.19 | 0.135 |
| Gender [n (%)] |  |  | 0.527 |
| Male | 3 (60%) | 2 (40%) |  |
| Female | 2 (40%) | 3 (60%) |  |
| Height (m) | 1.71 ± 0.01 | 1.62 ± 0.04 | 0.151 |
| Weight (kg) | 62.20 ± 1.77 | 57.0 ± 4.79 | 0.135 |
| BMI (kg/m^2^) | 21.33 ± 0.57 | 21.29 ± 0.90 | 0.841 |
| Smoking [n (%)] | 0 (0%) | 0 (0%) |  |

Data are shown as the Mean ± SEM or the number (percentage) for categorical data. Two-tailed non-parametric test (Mann-Whitney) or Chi-square test. IBS-D, Diarrhea-predominant irritable bowel syndrome; BMI, Body mass index.

**Supplementary Table S4: Relative expression of indole and its derivatives in the cecum**

|  | **Vehicle (veh)** | ***F. varium* (Fuso)** | ***P* value** | **Fuso *vs.* veh** |
| --- | --- | --- | --- | --- |
| 3-Methylene-indolenine | 109487907 ± 10106776 | 37764063 ± 7622447 | < 0.0001 | Down |
| Indole-3-acetic acid | 282154742 ± 28078174 | 91457135 ± 18637958 | < 0.0001 | Down |
| 2-Bromo-1H-indole-3-carboxaldehyde | 140493843 ± 28099035 | 44246770 ± 9757930 | 0.005 | Down |
| Indole-3-acrylic acid | 52554810 ± 3697589 | 67083906 ± 3747655 | 0.013 | Up |
| Methyl 5-hydroxyoxindole-3-acetate | 59095791 ± 6776983 | 117570860 ± 18918603 | 0.009 | Up |

Data are shown as the Mean ± SEM. Two-tailed non-parametric test (Mann-Whitney).

**Supplementary Table S5: Antibodies used in this study**

|  | **Host** | **Source** | **Identifier** | **Dilution** |
| --- | --- | --- | --- | --- |
| anti-Piezo2 antibody | Rabbit polyclonal | Prosci | CAT#8613 | 1:1000 (WB) |
| anti-Piezo2 antibody | Rabbit polyclonal | Novus | Cat# NBP1-78624 | 1:200-500 (IF) |
| anti-β-actin antibody | Mouse monoclonal | ZSBG-Bio | CAT#TA-09 | 1:1000 (WB) |
| IB_4_-FITC |  | Sigma-Aldrich | CAT#L2895 | 1:50 (IF) |
| anti-CGRP antibody | Mouse monoclonal | Abcam | CAT#ab81887 | 1:150 (IF) |
| anti-NF200 antibody | Mouse monoclonal | Sigma-Aldrich | CAT#N0142 | 1:200 (IF) |
| anti-TrkB antibody | Mouse monoclonal | Santa Cruz | CAT#sc136990 | 1:500 (IF) |
| anti-Serotonin antibody | Goat polyclonal | Immunostar | CAT#20097 | 1:1000 (IF) |
| anti-ChgA antibody | Mouse monoclonal | Immunoway | CAT#YM6617 | 1:400 (IF) |
| Hoechst |  | Solarbio | CAT#C0031 | 1:1000 (IF) |
| Alexa Fluor 488-conjugated donkey anti-rabbit | Rabbit polyclonal | Invitrogen | CAT# A21206 | 1:1000 (IF) |
| Alexa Fluor 594-conjugated donkey anti-mouse | Mouse polyclonal | Invitrogen | CAT#A21203 | 1:1000 (IF) |
| Alexa Fluor 594-conjugated donkey anti-goat | Goat polyclonal | Invitrogen | CAT#A11058 | 1:1000 (IF) |
| horseradish peroxidase (HRP)-conjugated goat anti-rabbit antibody |  | ZSBG-Bio | #ZB-2301 | 1:1000 (WB) |
| horseradish peroxidase (HRP)-conjugated goat anti-mouse antibody |  | ZSBG-Bio | #ZB-2305 | 1:1000 (WB) |

**Supplementary Figure 1.** Schematic diagram of IBS model established through Co-housing with FMT in pGF mice.

**Supplementary Figure 2.** The flowchart of the study.

**Supplementary Figure 3.** Reduced quality of life and intestinal dysbiosis in IBS-D patients.

**Supplementary Figure 4.** Gut microbiota analysis of FMT donors.

**Supplementary Figure 5.** Co-housing with FMT-IBS pGF mice induces visceral hypersensitivity, gut dysmotility and upregulates of Piezo2 in pGF mice.

**Supplementary Figure 6.** Body weight changes and colon H&E staining in colon or DRG Piezo2 KD mice.

**Supplementary Figure 7.** Phylum level composition of 16S rRNA sequencing in pGF mice after FMT.

**Supplementary Figure 8.** The supervised random forest analysis at the genus level.

**Supplementary Figure 9.** Comparative analysis of gut microbiota between donors and FMT-recipient mice.

**Supplementary Figure 10.** Oral gavage with *Fusobacterium varium*, rather than *Akkermansia muciniphila*, induced visceral hypersensitivity and gut dysmotility in pGF mice.

**Supplementary Figure11.** Upregulation of colon and DRG Piezo2 in the pGF mice following *Fusobacterium varium* intervention.

**Supplementary Figure 12.** *Fusobacterium varium* intervention did not alter weight change in colon or DRG Piezo2 knockdown pGF mice.

**Supplementary Figure 13.** Significant changes in the metabolome of pGF mice after intervention with *Fusobacterium varium*.

**Supplementary Figure 14.** Molecular docking suggests that indole-3-acetic acid and indole-3-acrylic acid have the potential to bind to Piezo2.
